# Supplementary figures and images for: Higher entropy observed in SARS-CoV-2 genomes from the first COVID-19 wave in Pakistan
Source: PLoS One. 2021 Aug 31;16(8):e0256451. doi: 10.1371/journal.pone.0256451 (PMC8407562; doi:10.1371/journal.pone.0256451)

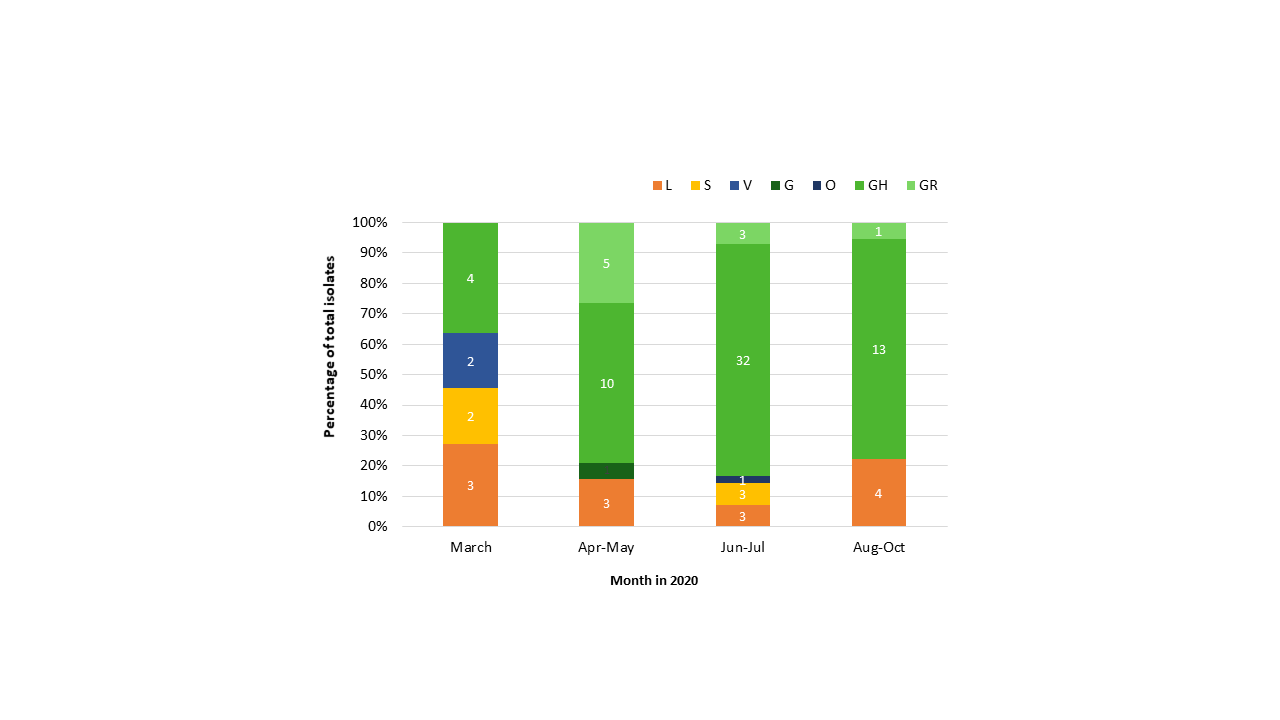

Supplement: S1 Fig — L, S, V, O and G, GH and GR clades are depicted as a percentage of the total genomes analysed in March (n = 11), April—May (n = 19), June—July (n = 42), and August—October (n = 18). The y axis represent the percentage of each isolate of the total number of strains. The number of SARS-CoV-2 strains of each clade within each period is inset within the graph. (TIF) [file pone.0256451.s001.tif]

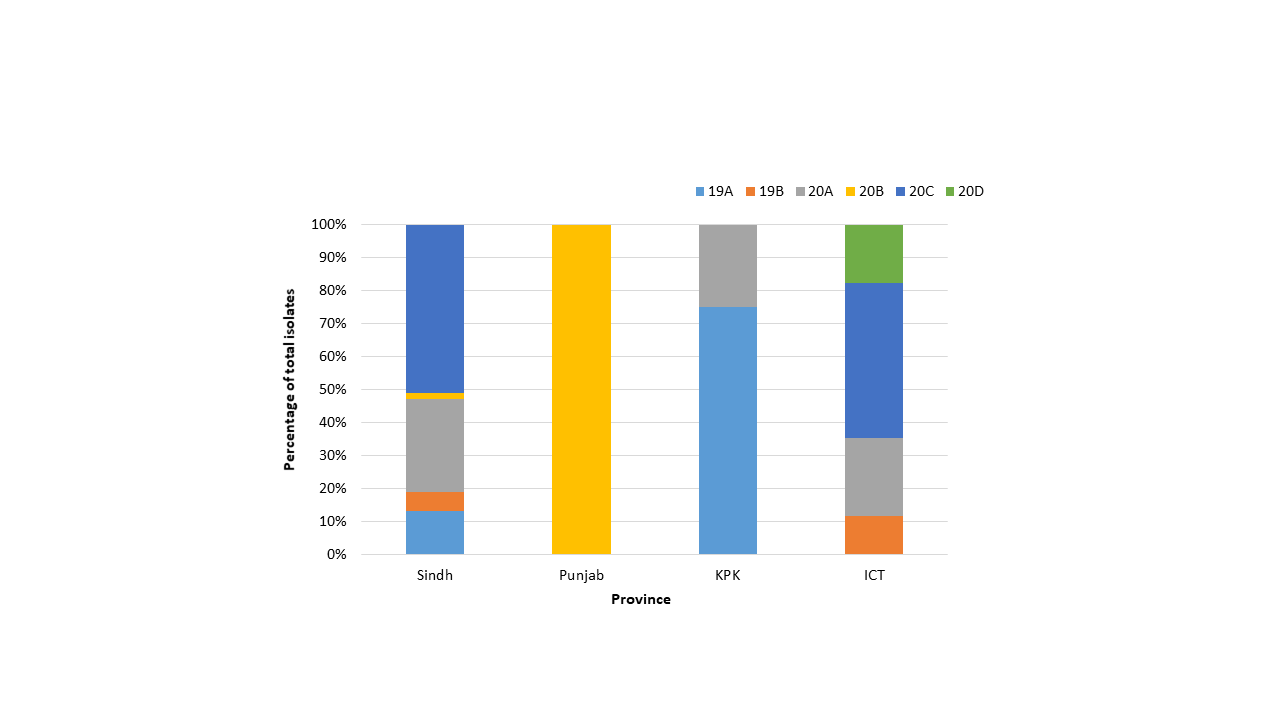

Supplement: S2 Fig — Next strain 19A,19B,20A, 20B, 20C and 20D clades are depicted as a percentage of the total genomes analysed from Sindh (n = 11), Punjab (n = 11), Khyber Pakhtunkhwa Province (KPK), n = 11 and Islamabad Capital Territory (ICT), n = 11. (TIF) [file pone.0256451.s002.tif]

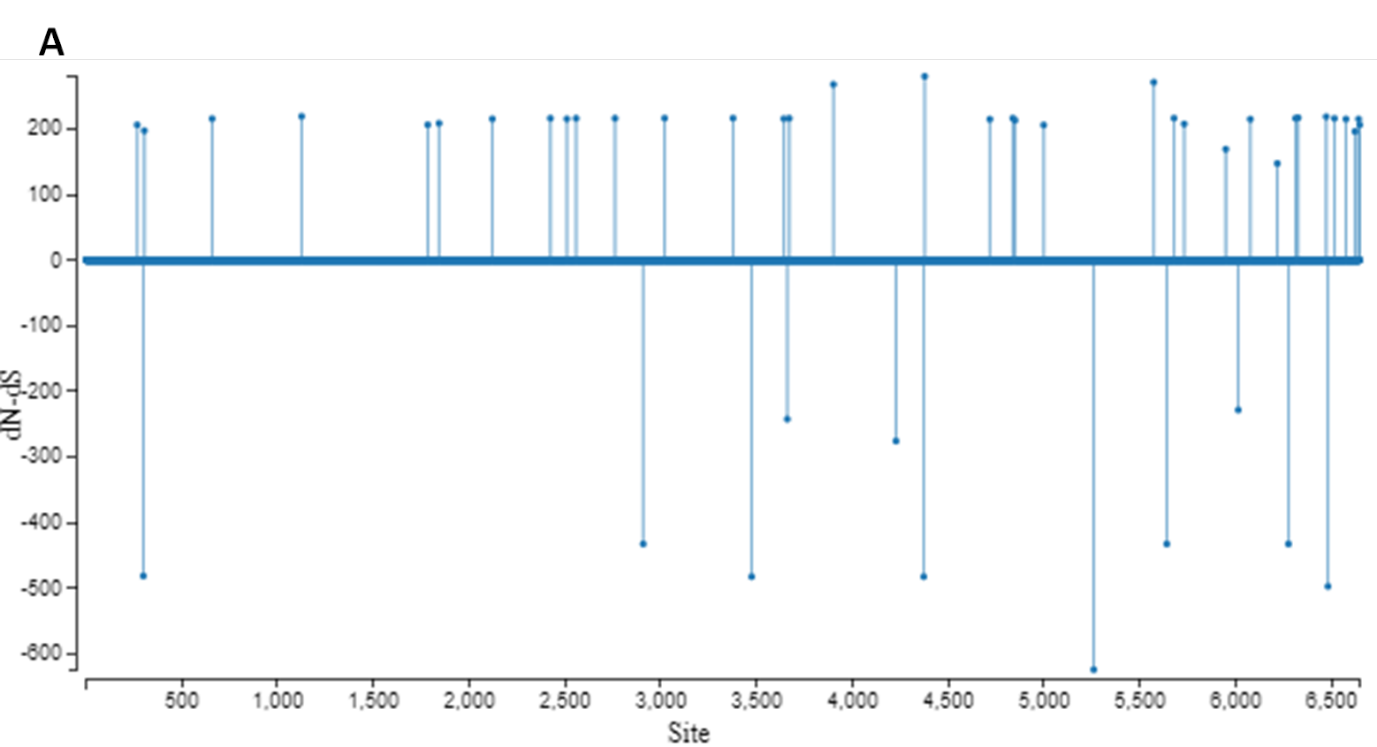

Supplement: S3 Fig — Selection pressure (relative number of non-synonymous substitutions minus synonymous substitutions (dN–dS)) on each codon is shown. (TIF) [file pone.0256451.s003.tif]

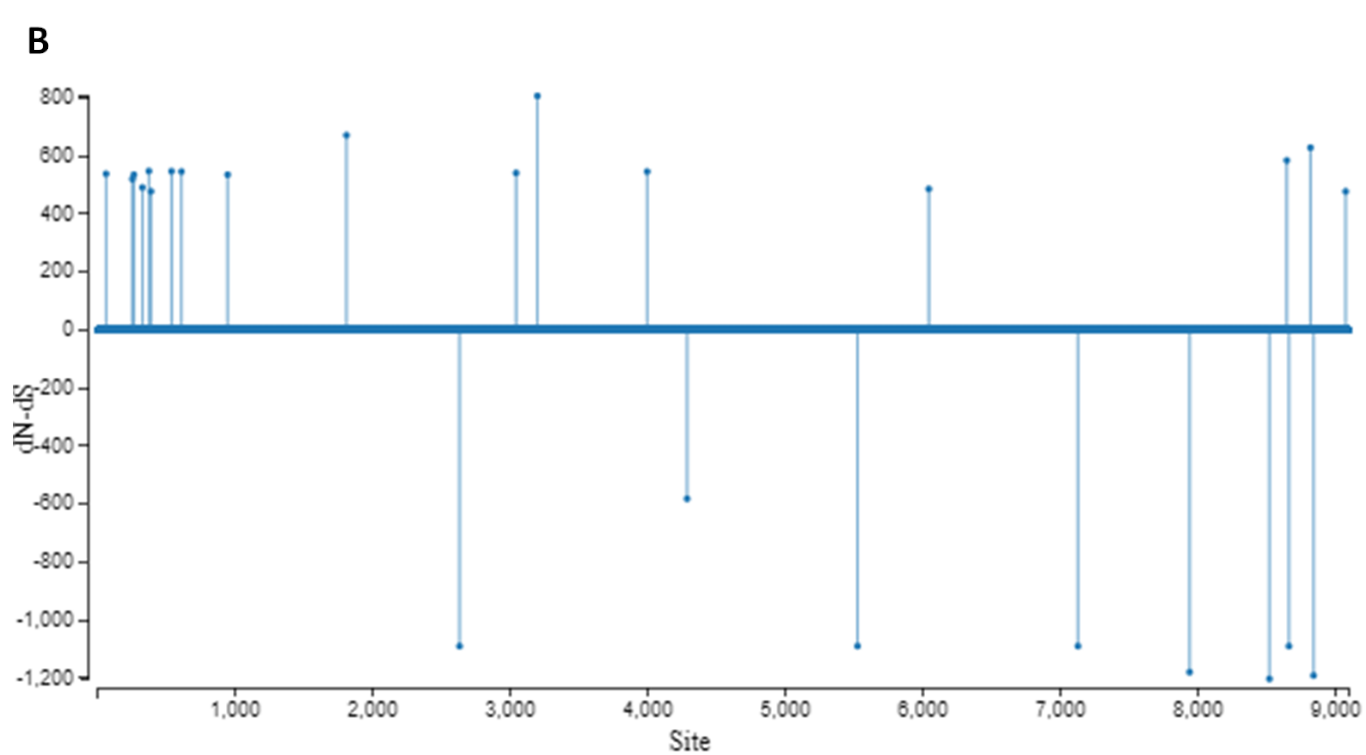

Supplement: S4 Fig — Selection pressure (relative number of non-synonymous substitutions minus synonymous substitutions (dN–dS)) on each codon is shown. (TIF) [file pone.0256451.s004.tif]

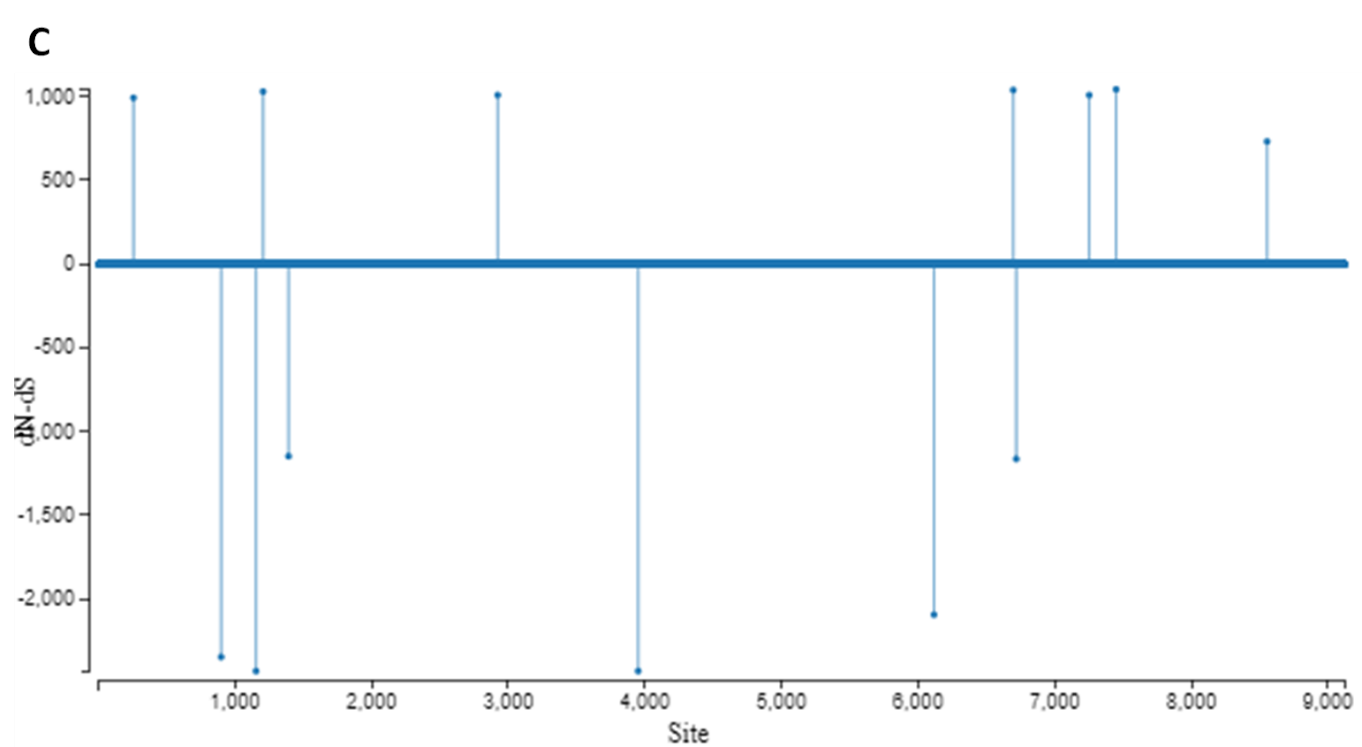

Supplement: S5 Fig — Selection pressure (relative number of non-synonymous substitutions minus synonymous substitutions (dN–dS)) on each codon is shown. (TIF) [file pone.0256451.s005.tif]
