# Supplementary material for: Higher entropy observed in SARS-CoV-2 genomes from the first COVID-19 wave in Pakistan
Source: PLoS One. 2021 Aug 31;16(8):e0256451. doi: 10.1371/journal.pone.0256451 (PMC8407562; doi:10.1371/journal.pone.0256451)
Supplement: S2 Table — (DOCX) [file pone.0256451.s007.docx]

**S2 Table. Description of variants found in 90 SARS-CoV-2 isolates from Pakistan.**

| **Sample IDs** | **No.** | **Position (+)** | **Type** | **Gene** | **Gene region** | **Amino acid Change** | **Nucleotide change** |
| --- | --- | --- | --- | --- | --- | --- | --- |
| hCoV19/Pakistan/NIH60/2020 | 1 | 98 | - | 5'UTR | 5'UTR | - | C > T |
| hCov-19/Pakistan/AKU-21/2020, hCov-19/Pakistan/AKU-2/2020, hCov-19/Pakistan/AKU-3/2020AKU, hCov-19/Pakistan/AKU-5/2020, hCoV-19/Pakistan/Gilgit1/2020, hCov-19/Pakistan/AKU-24/2020, hCov-19/Pakistan/AKU-25/2020, hCov-19/Pakistan/AKU-26/2020, hCov-19/Pakistan/AKU-27/2020, hCov-19/Pakistan/AKU-33/2020, hCov-19/Pakistan/AKU-37/2020, hCov-19/Pakistan/AKU-39/2020, hCov-19/Pakistan/AKU-46/2020, hCov-19/Pakistan/AKU-47/2020, hCov-19/Pakistan/AKU-54/2020, hCov-19/Pakistan/AKU-56/2020, hCov-19/Pakistan/AKU-64/2020, hCov-19/Pakistan/AKU-66/2020, hCov-19/Pakistan/AKU-67/2020, hCov-19/Pakistan/AKU-70/2020, hCov-19/Pakistan/AKU-52/2020, hCov-19/Pakistan/AKU-55/2020, PAK/NIH-45579/2020, PAK/NIH-44090/2020, PAK/NIH-44090/2020, hCoV-19/Pakistan/UN-UVAS-SIALKOT, hCoV-19/Pakistan/UN-UVAS-LAHORE-IV, hCoV-19/Pakistan/UN-UVAS-Lahore-III/2020, hCoV-19/Pakistan/UN-UVAS-Lahore-II/2020, hCoV-19/Pakistan/UN-UVAS-Lahore-IV/2020, hCoV-19/Pakistan/KPK-KUST-SJTU/2020, hCoV19/Pakistan/JRCGRKHI02/2020, hCoV19/Pakistan/JRCGRKHI03/2020, hCoV19/Pakistan/JRCGRKHI05/2020, hCoV19/Pakistan/JRCGRKHI06/2020, hCoV19/Pakistan/JRCGRKHI07/2020, hCoV19/Pakistan/JRCGRKHI11/2020, hCoV19/Pakistan/JRCGRKHI12/2020, hCoV19/Pakistan/JRCGRKHI13/2020, hCoV19/Pakistan/JRCGRKHI15/2020, hCoV19/Pakistan/JRCGRKHI16/2020, hCoV19/Pakistan/JRCGRKHI17/2020, hCoV19/Pakistan/JRCGRKHI18/2020, hCoV19/Pakistan/JRCGRKHI22/2020, hCoV19/Pakistan/JRCGRKHI06/2020, hCoV19/Pakistan/JRCGRKHI28/2020, hCoV19/Pakistan/JRCGRKHI29/2020, hCoV19/Pakistan/JRCGRKHI30/2020, hCoV19/Pakistan/JRCGRKHI32/2020, hCoV19/Pakistan/JRCGRKHI33/2020, hCoV19/Pakistan/JRCGRKHI37/2020, hCoV19/Pakistan/JRCGRKHI40/2020, hCoV19/Pakistan/JRCGRKHI41/2020, hCoV19/Pakistan/JRCGRKHI42/2020, hCoV19/Pakistan/JRCGRKHI45/2020, hCoV19/Pakistan/JRCGRKHI47/2020, hCoV19/Pakistan/JRCGRKHI48/2020, hCoV19/Pakistan/NIBD01KHI/2020, hCoV19/Pakistan/NIBD02KHI/2020, hCoV19/Pakistan/NIH60/2020, hCoV19/Pakistan/NIH64/2020, hCoV19/Pakistan/NIH68/2020, hCoV-19/Pakistan/NIH-76/2020, hCoV19/Pakistan/NIH78/2020 hCoV19/Pakistan/NIH95/2020, hCoV-19/Pakistan/NIH-66/2020, hCoV-19/Pakistan/NIH-70/2020, hCoV-19/Pakistan/NIH-62/2020, hCoV-19/Pakistan/NIH-65/2020, hCoV-19/Pakistan/JRCGR-KHI35/2020, hCoV-19/Pakistan/NIH-79/2020 | 71 | 241 | - | 5'UTR | 5'UTR | - | C > T |
| PAK/NIH-44905/2020 | 1 | 355 | Syn | Orf1ab | leader | p.30G | c.90ggC>ggT |
| hCoV19/Pakistan/JRCGRKHI33/2020 | 1 | 453 | Nsyn | Orf1ab | leader | p.63Q>L | c.188cAa>cTa |
| hCoV19/Pakistan/JRCGRKHI03/2020, hCoV19/Pakistan/JRCGRKHI13/2020 | 2 | 509 | Nsyn | Orf1ab | leader | p.82G>S | c.244Ggt>Agt |
| hCoV19/Pakistan/JRCGRKHI12/2020 | 1 | 611 | Nsyn | Orf1ab | leader | p.116V>L | c.346Gtg>Ttg |
| hCoV19/Pakistan/JRCGRKHI42/2020 | 1 | 829 | Syn | Orf1ab | nsp2 | p.188N | c.564aaC>aaT |
| hCoV19/Pakistan/JRCGRKHI04/2020 | 1 | 878 | Syn | Orf1ab | nsp2 | p.2839S | c.8517agC>agT |
| hCoV-19/Pakistan/Gilgit1/2020, hCov-19/Pakistan/AKU-58/2020, hCov-19/Pakistan/AKU-59/2020 | 3 | 884 | Nsyn | Orf1ab | nsp2 | p.R207C | c.619Cgt>Tgt |
| hCov-19/Pakistan/AKU-64/2020 | 1 | 896 | Nsyn | Orf1ab | nsp2 | p.211A>T | c.631Gct>Act |
| hCov-19/Pakistan/AKU-57/2020 | 1 | 920 | Syn | Orf1ab | nsp2 | p.219L | c.655Ctg>Ttg |
| hCov-19/Pakistan/AKU-3/2020 | 1 | 934 | Syn | Orf1ab | nsp2 | p.223D | c.669gaC>gaT |
| hCoV-19/Pakistan/NIH-70/2020, hCoV-19/Pakistan/NIH-65/2020 | 2 | 936 | Nsyn | Orf1ab | nsp2 | p.224T>I | C>T |
| hCoV19/Pakistan/JRCGRKHI11/2020, hCoV19/Pakistan/JRCGRKHI12/2020 | 2 | 1048 | Nsyn | Orf1ab | nsp2 | p.261K>N | c.783aaG>aaT |
| hCov-19/Pakistan/AKU-5/2020, hCov-19/Pakistan/AKU-70/2020 | 2 | 1059 | Nsyn | Orf1ab | nsp2 | p.265T>I | c.794aCc>aTc |
| hCov-19/Pakistan/AKU-56/2020 | 1 | 1082 | Nsyn | Orf1ab | nsp2 | p.273F>L | c.817Ttt>Ctt |
| hCoV19/Pakistan/JRCGRKHI28/2020, hCoV19/Pakistan/JRCGRKHI29/2020 | 2 | 1090 | Nsyn | Orf1ab | nsp2 | p.275F>L | c.825ttT>ttA |
| hCoV19/Pakistan/JRCGRKHI32/2020 | 1 | 1124 | Nsyn | Orf1ab | nsp2 | p.287R>G | c.859Agg>Ggg |
| hCoV-19/Pakistan/KP-RMI-01/2020 | 1 | 1139 | Nsyn | Orf1ab | nsp2 | p.292K>E | c.874Aag>Gag |
| hCov-19/Pakistan/AKU-47/2020 | 1 | 1267 | Syn | Orf1ab | nsp2 | p.334G | c.1002ggC>ggT |
| hCoV19/Pakistan/JRCGRKHI07/2020, hCoV19/Pakistan/JRCGRKHI15/2020, hCoV19/Pakistan/JRCGRKHI30/2020, hCoV19/Pakistan/JRCGRKHI45/2020 | 4 | 1351 | Syn | Orf1ab | nsp2 | p.362Q | c.1086caA>caG |
| hCoV-19/Pakistan/Gilgit1/2020 | 1 | 1384 | Syn | Orf1ab | nsp2 | p.361P | c.1083ccC>ccT |
| hCoV19/Pakistan/JRCGRKHI29/2020 | 1 | 1387 | Syn | Orf1ab | nsp2 | p.374H | c.1122caC>caT |
| hCov-19/Pakistan/AKU-58/2020, hCov-19/Pakistan/AKU-59/2020 | 3 | 1397 | Nsyn | Orf1ab | nsp2 | p.378V>I | c.1132Gta>Ata |
| hCov-19/Pakistan/AKU-54/2020 | 1 | 1545 | Nsyn | Orf1ab | nsp2 | p.427A>V | p.427A>V |
| hCoV-19/Pakistan/Gilgit1/2020 | 1 | 1397 | Nsyn | Orf1ab | nsp2 | p.V378I | c.1132Gta>Ata |
| PAK/NIH-45579/2020, hCoV19/Pakistan/NIH68/2020 | 2 | 1613 | Nsyn | Orf1ab | nsp2 | p.450L>F | c.1348Ctt>Ttt |
| hCoV-19/Pakistan/KHI1/2020, hCoV19/Pakistan/NIH64/2020, hCoV-19/Pakistan/NIH-62/2020 | 3 | 1912 | Syn | Orf1ab | nsp2 | p.549S | c.1647tcC>tcT |
| hCoV19/Pakistan/NIH64/2020 | 1 | 2032 | Syn | Orf1ab | nsp2 | p.589D | c.1767gaT>gaC |
| hCoV-19/Pakistan/KP-RMI-01/2020, hCoV19/Pakistan/JRCGRKHI15/2020, hCoV19/Pakistan/JRCGRKHI45/2020 | 3 | 2144 | Nsyn | Orf1ab | nsp2 | p.627V>F | c.1879Gtc>Ttc |
| hCov-19/Pakistan/AKU-21/2020, hCov-19/Pakistan/AKU-67/2020, PAK/NIH-45579/2020, PAK/NIH-44090/2020, PAK/NIH-45143/2020, hCoV-19/Pakistan/KPK-KUST-SJTU/2020, hCoV19/Pakistan/JRCGRKHI22/2020, hCoV19/Pakistan/JRCGRKHI32/2020, hCoV19/Pakistan/NIH68/2020, hCoV-19/Pakistan/NIH-76/2020, hCoV19/Pakistan/NIH78/2020, hCoV19/Pakistan/NIH95/2020 | 12 | 2416 | Syn | Orf1ab | nsp2 | p.717Y | c.2151taC>taT |
| hCov-19/Pakistan/AKU-47/2020 | 1 | 2432 | Nsyn | Orf1ab | nsp2 | p.723S>T | c.2167Tcc>Acc |
| PAK/NIH-HAS001/2020, PAK/NIH-44905/2020 | 2 | 2461 | Syn | Orf1ab | nsp2 | p.732P | c.2196ccT>ccC |
| hCoV19/Pakistan/NIH60/2020 | 1 | 2607 | Nsyn | Orf1ab | nsp2 | p.781T>I | c.2342aCa>aTa |
| hCov-19/Pakistan/AKU-55/2020 | 1 | 2749 | Syn | Orf1ab | nsp3 | p.843F | c.2529ttT>ttC |
| hCov-19/Pakistan/AKU-47/2020 | 1 | 2973 | Nsyn | Orf1ab | nsp3 | p.903A>V | c.2708gCt>gTt |
| hCov-19/Pakistan/AKU-21/2020, hCov-19/Pakistan/AKU-2/2020, hCov-19/Pakistan/AKU-3/2020, hCov-19/Pakistan/AKU-5/2020, hCov-19/Pakistan/AKU-24/2020, hCov-19/Pakistan/AKU-25/2020, hCov-19/Pakistan/AKU-26/2020, hCov-19/Pakistan/AKU-27/2020, hCov-19/Pakistan/AKU-33/2020, hCov-19/Pakistan/AKU-37/2020, hCov-19/Pakistan/AKU-39/2020, hCov-19/Pakistan/AKU-46/2020, hCov-19/Pakistan/AKU-47/2020, hCov-19/Pakistan/AKU-54/2020, hCov-19/Pakistan/AKU-56/2020, hCov-19/Pakistan/AKU-64/2020, hCov-19/Pakistan/AKU-66/2020, hCov-19/Pakistan/AKU-67/2020, hCov-19/Pakistan/AKU-70/2020, PAK/NIH-45579/2020, PAK/NIH-44090/2020, PAK/NIH-45143/2020, hCoV-19/Pakistan/UN-UVAS-SIALKOT, hCoV-19/Pakistan/UN-UVAS-LAHORE-IV, hCoV-19/Pakistan/UN-UVAS-Lahore-III/2020, hCoV-19/Pakistan/UN-UVAS-Lahore-II/2020, hCoV-19/Pakistan/UN-UVAS-Lahore-IV/2020, hCoV-19/Pakistan/KPK-KUST-SJTU/2020, hCoV19/Pakistan/JRCGRKHI02/2020, hCoV19/Pakistan/JRCGRKHI03/2020, hCoV19/Pakistan/JRCGRKHI05/2020, hCoV19/Pakistan/JRCGRKHI06/2020, hCoV19/Pakistan/JRCGRKHI07/2020, hCoV19/Pakistan/JRCGRKHI11/2020, hCoV19/Pakistan/JRCGRKHI12/2020, hCoV19/Pakistan/JRCGRKHI13/2020, hCoV19/Pakistan/JRCGRKHI15/2020, hCoV19/Pakistan/JRCGRKHI16/2020, hCoV19/Pakistan/JRCGRKHI17/2020, hCoV19/Pakistan/JRCGRKHI22/2020, hCoV19/Pakistan/JRCGRKHI06/2020, hCoV19/Pakistan/JRCGRKHI28/2020, hCoV19/Pakistan/JRCGRKHI29/2020, hCoV19/Pakistan/JRCGRKHI30/2020, hCoV19/Pakistan/JRCGRKHI32/2020, hCoV19/Pakistan/JRCGRKHI33/2020, hCoV19/Pakistan/JRCGRKHI37/2020, hCoV19/Pakistan/JRCGRKHI40/2020, hCoV19/Pakistan/JRCGRKHI41/2020, hCoV19/Pakistan/JRCGRKHI42/2020, hCoV19/Pakistan/JRCGRKHI45/2020, hCoV19/Pakistan/JRCGRKHI47/2020, hCoV19/Pakistan/JRCGRKHI48/2020, hCoV19/Pakistan/NIBD01KHI/2020, hCoV19/Pakistan/NIBD02KHI/2020, hCoV19/Pakistan/NIH60/2020, hCoV19/Pakistan/NIH64/2020, hCoV19/Pakistan/NIH68/2020, hCoV-19/Pakistan/NIH-76/2020, hCoV19/Pakistan/NIH78/2020, hCoV19/Pakistan/NIH95/2020, hCoV-19/Pakistan/NIH-66/2020, hCoV-19/Pakistan/NIH-70/2020, hCoV-19/Pakistan/NIH-62/2020, hCoV-19/Pakistan/NIH-65/2020, hCoV-19/Pakistan/JRCGR-KHI35/2020, hCoV-19/Pakistan/NIH-79/2020 | 67 | 3037 | Syn | Orf1ab | nsp3 | p.924F | c.2772ttC>ttT |
| hCoV19/Pakistan/JRCGRKHI07/2020 | 1 | 3065 | Nsyn | Orf1ab | nsp3 | p.934G>C | c.2800Ggt>Tgt |
| hCoV-19/Pakistan/UN-UVAS-LAHORE-IV, hCoV-19/Pakistan/UN-UVAS-Lahore-III/2020, hCoV-19/Pakistan/UN-UVAS-Lahore-II/2020, hCoV-19/Pakistan/UN-UVAS-Lahore-IV/2020 | 4 | 3096 | Nsyn | Orf1ab | nsp3 | p.944S>L | c.2831tCa>tTa |
| hCoV19/Pakistan/JRCGRKHI30/2020, hCoV-19/Pakistan/NIH-70/2020, hCoV-19/Pakistan/NIH-65/2020 | 3 | 3261 | Nsyn | Orf1ab | nsp3 | p.999T>I | c.2996aCa>aTa |
| hCov-19/Pakistan/AKU-57/2020 | 1 | 3311 | Nsyn | Orf1ab | nsp3 | p.1016L>F | c.3046Ctt>Ttt |
| hCov-19/Pakistan/AKU-58/2020, hCov-19/Pakistan/AKU-59/2020 | 2 | 3330 | Nsyn | Orf1ab | nsp3 | p.1022T>I | c.3065aCt>aTt |
| PAK/NIH-45579/2020 | 1 | 3613 | Syn | Orf1ab | nsp3 | p.1116H | c.3348caT>caC |
| hCoV19/Pakistan/NIH60/2020 | 1 | 3743 | Nsyn | Orf1ab | nsp3 | p.1160H>Y | p.1160H>Y |
| hCoV19/Pakistan/JRCGRKHI05/2020 | 1 | 3788 | Nsyn | Orf1ab | nsp3 | p.1175L>I | c.3523Tta>Ata |
| hCov-19/Pakistan/AKU-56/2020 | 1 | 3898 | Syn | Orf1ab | nsp3 | p.1211V | c.3633gtT>gtC |
| hCoV-19/Pakistan/UN-UVAS-LAHORE-IV, hCoV-19/Pakistan/UN-UVAS-Lahore-III/2020, hCoV-19/Pakistan/UN-UVAS-Lahore-II/2020, hCoV-19/Pakistan/UN-UVAS-Lahore-IV/2020, hCoV-19/Pakistan/NIH-62/2020, hCoV-19/Pakistan/NIH-79/2020 | 6 | 4002 | Nsyn | Orf1ab | nsp3 | p.1246T>I | c.3737aCt>aTt |
| hCoV19/Pakistan/NIBD01KHI/2020, hCoV19/Pakistan/NIBD02KHI/2020 | 2 | 4126 | Syn | Orf1ab | nsp3 | p.1287V | c.3861gtG>gtA |
| hCoV-19/Pakistan/NIH-70/2020, hCoV-19/Pakistan/NIH-65/2020 | 2 | 4160 | Nsyn | Orf1ab | nsp3 | p.1299V>L | G>T |
| hCoV-19/Pakistan/UN-UVAS-LAHORE-IV, hCoV-19/Pakistan/UN-UVAS-Lahore-III/2020, hCoV-19/Pakistan/UN-UVAS-Lahore-II/2020, hCoV-19/Pakistan/UN-UVAS-Lahore-IV/2020 | 4 | 4180 | Nsyn | Orf1ab | nsp3 | p.1305K>N | c.3915aaG>aaT |
| hCov-19/Pakistan/AKU-56/2020 | 1 | 4414 | Syn | Orf1ab | nsp3 | p.1383E | c.4149gaA>gaG |
| hCoV19/Pakistan/JRCGRKHI48/2020 | 1 | 4720 | Syn | Orf1ab | nsp3 | p.1485A | c.4455gcG>gcT |
| hCov-19/Pakistan/AKU-64/2020, hCoV19/Pakistan/NIH64/2020 | 2 | 4795 | Syn | Orf1ab | nsp3 | p.1510S | c.4530tcC>tcT |
| hCov-19/Pakistan/AKU-65/2020 | 1 | 5120 | Nsyn | Orf1ab | nsp3 | p.1619Y>H | c.4855Tat>Cat |
| hCov-19/Pakistan/AKU-52/2020 | 1 | 5209 | Syn | Orf1ab | nsp3 | p.1648S | c.4944tcA>tcG |
| hCoV19/Pakistan/NIBD01KHI/2020, hCoV19/Pakistan/NIBD02KHI/2020 | 2 | 5482 | Syn | Orf1ab | nsp3 | p.1739A | c.5217gcC>gcT |
| hCoV19/Pakistan/JRCGRKHI42/2020 | 1 | 5526 | Nsyn | Orf1ab | nsp3 | p.1754T>I | c.5261aCt>aTt |
| hCoV19/Pakistan/JRCGRKHI15/2020, hCoV19/Pakistan/JRCGRKHI45/2020 | 2 | 6040 | Syn | Orf1ab | nsp3 | p.1925F | c.5775ttC>ttT |
| hCoV19/Pakistan/JRCGRKHI40/2020 | 1 | 6042 | Nsyn | Orf1ab | nsp3 | p.1926D>G | c.5777gAt>gGt |
| hCov-19/Pakistan/AKU-23/2020, hCov-19/Pakistan/AKU-57/2020, hCoV-19/Pakistan/KP-RMI-01/2020 | 3 | 6312 | Nsyn | Orf1ab | nsp3 | p.2016T>K | c.6047aCa>aAa |
| hCov-19/Pakistan/AKU-21/2020 | 1 | 6532 | Nsyn | Orf1ab | nsp3 | p.2089E>D | c.6267gaG>gaT |
| hCoV19/Pakistan/JRCGRKHI17/2020, hCoV19/Pakistan/JRCGRKHI28/2020, hCoV19/Pakistan/JRCGRKHI29/2020 | 3 | 6663 | Nsyn | Orf1ab | nsp3 | p.2133V>A | c.6398gTc>gCc |
| hCoV19/Pakistan/JRCGRKHI17/2020, hCoV19/Pakistan/JRCGRKHI18/2020 | 2 | 7488 | Nsyn | Orf1ab | nsp3 | p.2408T>I | c.7223aCt>aTt |
| hCoV19/Pakistan/JRCGRKHI03/2020 | 1 | 7955 | Nsyn | Orf1ab | nsp3 | p.2564L>F | c.7690Ctt>Ttt |
| hCov-19/Pakistan/AKU-54/2020, hCoV19/Pakistan/JRCGRKHI41/2020 | 1 | 8118 | Nsyn | Orf1ab | nsp3 | p.2618A>V | c.7853gCt>gTt |
| hCov-19/Pakistan/AKU-21/2020, hCov-19/Pakistan/AKU-67/2020, PAK/NIH-45579/2020, PAK/NIH-44090/2020, PAK/NIH-45143/2020, hCoV-19/Pakistan/KPK-KUST-SJTU/2020, hCoV19/Pakistan/JRCGRKHI22/2020, hCoV19/Pakistan/JRCGRKHI32/2020, hCoV19/Pakistan/NIH68/2020, hCoV-19/Pakistan/NIH-76/2020, hCoV19/Pakistan/NIH78/2020, hCoV19/Pakistan/NIH95/2020 | 12 | 8371 | Nsyn | Orf1ab | nsp3 | p.2702Q>H | c.8106caG>caT |
| hCov-19/Pakistan/AKU-58/2020, hCov-19/Pakistan/AKU-59/2020 | 2 | 8653 | Nsyn | Orf1ab | nsp4 | p.2796M>I | c.8388atG>atT |
| hCov-19/Pakistan/AKU-10/2020, hCov-19/Pakistan/AKU-11/2020, PAK/NIH-HAS001/2020, PAK/NIH-44905/2020 | 4 | 8782 | Syn | Orf1ab | nsp4 | p.2839S | c.8517agC>agT |
| hCoV19/Pakistan/JRCGRKHI42/2020 | 1 | 8802 | Nsyn | Orf1ab | nsp4 | p.2846T>I | c.8537aCt>aTt |
| hCov-19/Pakistan/AKU-39/2020 | 1 | 8812 | Syn | Orf1ab | nsp4 | p.2849K | c.8547aaA>aaG |
| hCoV-19/Pakistan/Gilgit1/2020 | 1 | 8894 | Nsyn | Of1ab | nsp4 | p.P2965L | c.8894cCt>cTt |
| hCov-19/Pakistan/AKU-37/2020 | 1 | 9042 | Nsyn | Orf1ab | nsp4 | p.2926S>Y | c.8777tCt>tAt |
| hCoV-19/Pakistan/KPK-KUST-SJTU/2020 | 1 | 9208 | Syn | Orf1ab | nsp4 | p.2981S | c.8943tcT>tcC |
| hCoV-19/Pakistan/NIH-70/2020, hCoV-19/Pakistan/NIH-65/2020 | 2 | 9214 | Syn | Orf1ab | nsp4 | p.2984C | C>T |
| hCoV-19/Pakistan/UN-UVAS-LAHORE-IV, hCoV-19/Pakistan/UN-UVAS-Lahore-III/2020, hCoV-19/Pakistan/UN-UVAS-Lahore-II/2020, hCoV-19/Pakistan/NIH-62/2020, hCoV-19/Pakistan/NIH-79/2020 | 5 | 10097 | Nsyn | Orf1ab | 3C-like proteinase | p.3278G>S | c.9832Ggt>Agt |
| hCoV19/Pakistan/JRCGRKHI33/2020 | 1 | 10142 | Nsyn | Orf1ab | 3C-like proteinase | p.3293L>F | c.9877Ctt>Ttt |
| hCov-19/Pakistan/AKU-47/2020, hCoV-19/Pakistan/UN-UVAS-Lahore-IV/2020 | 2 | 10279 | Syn | Orf1ab | 3C-like proteinase | p.3338L | c.10014ctC>ctT |
| hCoV19/Pakistan/JRCGRKHI22/2020 | 1 | 10323 | Syn | Orf1ab | 3C-like proteinase | p.3353K>R | c.10058aAg>aGg |
| hCoV-19/Pakistan/KHI1/2020 | 1 | 10582 | Syn | Orf1ab | 3C-like proteinase | p.3439D | 10317gaC>gaT |
| hCov-19/Pakistan/AKU-54/2020, hCov-19/Pakistan/AKU-50/2020 | 2 | 10369 | Syn | Orf1ab | 3C-like proteinase | p.3368R | c.10104cgC>cgT |
| hCoV19/Pakistan/JRCGRKHI48/2020 | 1 | 10604 | Nsyn | Orf1ab | 3C-like proteinase | p.3447P>S | c.10339Cct>Tct |
| hCov-19/Pakistan/AKU-54/2020 | 1 | 10626 | Nsyn | Orf1ab | 3C-like proteinase | p.3454A>V | c.10361gCa>gTa |
| hCoV19/Pakistan/JRCGRKHI12/2020 | 1 | 10688 | Nsyn | Orf1ab | 3C-like proteinase | p.3475V>F | c.10423Gtt>Ttt |
| PAK/NIH-45143/2020 | 1 | 10738 | Nsyn | Orf1ab | 3C-like proteinase | p.3491N>K | c.10473aaT>aaA |
| hCoV-19/Pakistan/KPK-KUST-SJTU/2020 | 1 | 10741 | Syn | Orf1ab | 3C-like proteinase | p.3492D | c.10476gaC>gaT |
| hCov-19/Pakistan/AKU-66/2020 | 1 | 11074 | Nsyn | Orf1ab | nsp6 | p.3603-3604->X | c.10809-10810->T |
| hCov-19/Pakistan/AKU-21/2020, hCov-19/Pakistan/AKU-23/2020, hCov-19/Pakistan/AKU-57/2020, hCov-19/Pakistan/AKU-58/2020, hCov-19/Pakistan/AKU-59/2020, PAK/NIH-45579/2020, PAK/NIH-44090/2020, PAK/NIH-45143/2020, hCoV-19/Pakistan/KPK-KUST-SJTU/2020, hCoV19/Pakistan/JRCGRKHI32/2020, hCoV19/Pakistan/NIH68/2020, hCoV-19/Pakistan/NIH-76/2020, hCoV19/Pakistan/NIH78/2020, hCoV19/Pakistan/NIH95/2020 | 14 | 11083 | Nsyn | Orf1ab | nsp6 | p.3606L>F | c.10818ttG>ttT |
| hCoV19/Pakistan/JRCGRKHI15/2020, hCoV19/Pakistan/JRCGRKHI45/2020 | 2 | 11201 | Nsyn | Orf1ab | nsp6 | p.3646T>A | c.10936Act>Gct |
| hCov-19/Pakistan/AKU-24/2020 | 3 | 11222 | Nsyn | Orf1ab | nsp6 | p.3653V>F | c.10957Gtc>Ttc |
| PAK/NIH-HAS001/2020, PAK/NIH-44905/2020 | 2 | 11230 | Nsyn | Orf1ab | nsp6 | p.3655M>I | c.10965atG>atT |
| hCoV19/Pakistan/JRCGRKHI32/2020 | 1 | 11234 | Nsyn | Orf1ab | nsp6 | p.3657A>T | c.10969Gct>Act |
| hCoV-19/Pakistan/NIH-70/2020, hCoV-19/Pakistan/NIH-65/2020 | 2 | 11668 | Syn | Orf1ab | nsp6 | p.3801N | C>T |
| hCoV19/Pakistan/NIH60/2020 | 1 | 11758 | Syn | Orf1ab | nsp6 | p.3831P | c.11493ccC>ccT |
| hCov-19/Pakistan/AKU-47/2020 | 1 | 12049 | Syn | Orf1ab | nsp7 | p.3928N | c.11784aaC>aaT |
| hCov-19/Pakistan/AKU-47/2020 | 1 | 12115 | Syn | Orf1ab | nsp8 | p.3950S | c.11850tcC>tcT |
| hCoV19/Pakistan/JRCGRKHI37/2020, hCoV19/Pakistan/JRCGRKHI47/2020 hCoV-19/Pakistan/JRCGR-KHI35/2020, | 3 | 12132 | Nsyn | Orf1ab | nsp8 | p.3956A>V | c.11867gCt>gTt |
| hCoV19/Pakistan/NIH60/2020 | 1 | 12400 | Syn | Orf1ab | nsp8 | p.4045L | c.12135ctC>ctT |
| hCoV19/Pakistan/JRCGRKHI33/2020 | 1 | 12488 | Nsyn | Orf1ab | nsp8 | p.4075P>S | c.12223Cca>Tca |
| hCov-19/Pakistan/AKU-56/2020 | 1 | 12534 | Nsyn | Orf1ab | nsp8 | p.4090T>I | c.12269aCt>aTt |
| hCoV-19/Pakistan/KPK-KUST-SJTU/2020 | 1 | 12565 | Syn | Orf1ab | nsp8 | p.4100Q | c.12300caG>caA |
| hCov-19/Pakistan/AKU-46/2020 | 1 | 12737 | Nsyn | Orf1ab | nsp9 | p.4158T>A | c.12472Act>Gct |
| hCoV19/Pakistan/JRCGRKHI04/2020 | 1 | 12970 | Syn | Orf1ab | nsp9 | p.4235N | c.12705aaC>aaT |
| hCoV-19/Pakistan/UN-UVAS-LAHORE-IV, hCoV-19/Pakistan/UN-UVAS-Lahore-III/2020, hCoV-19/Pakistan/UN-UVAS-Lahore-II/2020, hCoV-19/Pakistan/UN-UVAS-Lahore-IV/2020, hCoV-19/Pakistan/NIH-62/2020, hCoV-19/Pakistan/NIH-79/2020 | 6 | 13536 | Syn | Orf1ab | RdRp | p.4424Y | c.13272taC>taT |
| hCov-19/Pakistan/AKU-56/2020 | 1 | 13724 | Nsyn | Orf1ab | RdRp | p.4487A>V | c.13460gCt>gTt |
| hCov-19/Pakistan/AKU-23/2020, hCov-19/Pakistan/AKU-57/2020, hCoV-19/Pakistan/KP-RMI-01/2020 | 3 | 13730 | Nsyn | Orf1ab | RdRp | p.4489A>V | c.13466gCt>gTt |
| hCov-19/Pakistan/AKU-51/2020, hCov-19/Pakistan/AKU-63/2020 | 2 | 13813 | Nsyn | Orf1ab | RdRp | p.4517A>T | c.13549Gca>Aca |
| hCov-19/Pakistan/AKU-24/2020 | 1 | 14184 | Syn | Orf1ab | RdRp | p.4640T | c.13920acC>acT |
| hCoV19/Pakistan/JRCGRKHI33/2020 | 1 | 14358 | Syn | Orf1ab | RdRp | p.4698C | c.14094tgC>tgT |
| hCov-19/Pakistan/AKU-21/2020, hCov-19/Pakistan/AKU-2/2020, hCov-19/Pakistan/AKU-3/2020, hCov-19/Pakistan/AKU-5/2020, hCov-19/Pakistan/AKU-24/2020, hCov-19/Pakistan/AKU-25/2020, hCov-19/Pakistan/AKU-26/2020, hCov-19/Pakistan/AKU-27/2020, hCov-19/Pakistan/AKU-33/2020, hCov-19/Pakistan/AKU-37/2020, hCov-19/Pakistan/AKU-39/2020, hCov-19/Pakistan/AKU-46/2020, hCov-19/Pakistan/AKU-47/2020, hCov-19/Pakistan/AKU-54/2020, hCov-19/Pakistan/AKU-56/2020, hCov-19/Pakistan/AKU-64/2020, hCov-19/Pakistan/AKU-66/2020, hCov-19/Pakistan/AKU-67/2020, hCov-19/Pakistan/AKU-70/2020, PAK/NIH-45579/2020, PAK/NIH-44090/2020, PAK/NIH-45143/2020, hCoV-19/Pakistan/UN-UVAS-SIALKOT, hCoV-19/Pakistan/UN-UVAS-LAHORE-IV, hCoV-19/Pakistan/UN-UVAS-Lahore-III/2020, hCoV-19/Pakistan/UN-UVAS-Lahore-II/2020, hCoV-19/Pakistan/UN-UVAS-Lahore-IV/2020, hCoV-19/Pakistan/KPK-KUST-SJTU/2020, hCoV19/Pakistan/JRCGRKHI02/2020, hCoV19/Pakistan/JRCGRKHI03/2020, hCoV19/Pakistan/JRCGRKHI05/2020, hCoV19/Pakistan/JRCGRKHI07/2020, hCoV19/Pakistan/JRCGRKHI11/2020, hCoV19/Pakistan/JRCGRKHI12/2020, hCoV19/Pakistan/JRCGRKHI13/2020, hCoV19/Pakistan/JRCGRKHI15/2020, hCoV19/Pakistan/JRCGRKHI16/2020, hCoV19/Pakistan/JRCGRKHI17/2020, hCoV19/Pakistan/JRCGRKHI22/2020, hCoV19/Pakistan/JRCGRKHI28/2020, hCoV19/Pakistan/JRCGRKHI29/2020, hCoV19/Pakistan/JRCGRKHI30/2020, hCoV19/Pakistan/JRCGRKHI32/2020, hCoV19/Pakistan/JRCGRKHI33/2020, hCoV19/Pakistan/JRCGRKHI37/2020, hCoV19/Pakistan/JRCGRKHI40/2020, hCoV19/Pakistan/JRCGRKHI41/2020, hCoV19/Pakistan/JRCGRKHI42/2020, hCoV19/Pakistan/JRCGRKHI45/2020, hCoV19/Pakistan/JRCGRKHI47/2020, hCoV19/Pakistan/JRCGRKHI48/2020, hCoV19/Pakistan/NIBD01KHI/2020, hCoV19/Pakistan/NIBD02KHI/2020, hCoV19/Pakistan/NIH60/2020, hCoV19/Pakistan/NIH64/2020, hCoV19/Pakistan/NIH68/2020, hCoV-19/Pakistan/NIH-76/2020, hCoV19/Pakistan/NIH78/2020, hCoV19/Pakistan/NIH95/2020, hCoV-19/Pakistan/NIH-66/2020, hCoV-19/Pakistan/NIH-70/2020, hCoV-19/Pakistan/NIH-62/2020, hCoV-19/Pakistan/NIH-65/2020, hCoV-19/Pakistan/JRCGR-KHI35/2020, hCoV-19/Pakistan/NIH-79/2020 | 65 | 14408 | Nsyn | Orf1ab | RdRp | p.4715P>L | c.14144cCt>cTt |
| hCoV19/Pakistan/JRCGRKHI15/2020, hCoV19/Pakistan/JRCGRKHI18/2020, hCoV19/Pakistan/JRCGRKHI45/2020 | 3 | 14685 | Syn | Orf1ab | RdRp | p.4807F | c.14421ttT>ttC |
| hCov-19/Pakistan/AKU-27/2020 | 1 | 14585 | Nsyn | Orf1ab | RdRp | p.4774A>V | c.14321gCt>gTt |
| hCov-19/Pakistan/AKU-57/2020 | 1 | 14599 | Syn | Orf1ab | RdRp | p.4779L | c.14335Cta>Tta |
| hCoV19/Pakistan/JRCGRKHI03/2020, hCoV19/Pakistan/JRCGRKHI13/2020 | 3 | 15026 | Nsyn | Orf1ab | RdRp | p.4921A>V | c.14762gCa>gTa |
| hCoV19/Pakistan/JRCGRKHI22/2020 | 1 | 15199 | Nsyn | Orf1ab | RdRp | p.4979V>L | c.14935Gta>Tta |
| hCov-19/Pakistan/AKU-25/2020 | 1 | 15237 | Syn | Orf1ab | RdRp | p.4991H | c.14973caC>caT |
| hCoV19/Pakistan/JRCGRKHI15/2020, hCoV19/Pakistan/JRCGRKHI45/2020 | 2 | 15348 | Syn | Orf1ab | RdRp | p.5028L | c.15084ctT>ctC |
| hCoV19/Pakistan/JRCGRKHI41/2020 | 1 | 15763 | Syn | Orf1ab | RdRp | p.5167L | c.15499Cta>Tta |
| hCov-19/Pakistan/AKU-47/2020 | 1 | 15854 | Nsyn | Orf1ab | RdRp | p.5197L>P | c.15590cTt>cCt |
| hCov-19/Pakistan/AKU-37/2020 | 1 | 16308 | Syn | Orf1ab | helicase | p.5348F | c.16044ttC>ttT |
| hCoV19/Pakistan/JRCGRKHI11/2020, hCoV19/Pakistan/JRCGRKHI12/2020 | 2 | 16616 | Nsyn | Orf1ab | helicase | p.5451T>I | c.16352aCt>aTt |
| hCov-19/Pakistan/AKU-27/2020 | 1 | 16861 | Nsyn | Orf1ab | helicase | p.5533V>F | c.16597Gtt>Ttt |
| hCov-19/Pakistan/AKU-25/2020, hCov-19/Pakistan/AKU-26/2020, hCov-19/Pakistan/AKU-33/2020, hCov-19/Pakistan/AKU-54/2020, hCov-19/Pakistan/AKU-56/2020, hCov-19/Pakistan/AKU-66/2020, hCoV19/Pakistan/JRCGRKHI06/2020, hCoV19/Pakistan/JRCGRKHI07/2020, hCoV19/Pakistan/JRCGRKHI12/2020, hCoV19/Pakistan/JRCGRKHI15/2020, hCoV19/Pakistan/JRCGRKHI16/2020, hCoV19/Pakistan/JRCGRKHI06/2020, hCoV19/Pakistan/JRCGRKHI30/2020, hCoV19/Pakistan/JRCGRKHI33/2020, hCoV19/Pakistan/JRCGRKHI40/2020, hCoV19/Pakistan/JRCGRKHI41/2020, hCoV19/Pakistan/JRCGRKHI42/2020, hCoV19/Pakistan/JRCGRKHI45/2020, hCoV19/Pakistan/JRCGRKHI48/2020, hCoV19/Pakistan/NIH60/2020 | 20 | 16915 | Syn | Orf1ab | helicase | p.5551L | c.16651Ctg>Ttg |
| hCoV-19/Pakistan/KPK-KUST-SJTU/2020 | 1 | 16945 | Nsyn | Orf1ab | helicase | p.5561A>T | c.16681Gca>Aca |
| hCoV19/Pakistan/JRCGRKHI30/2020 | 1 | 17004 | Syn | Orf1ab | helicase | p.5580L | c.16740ctC>ctT |
| hCov-19/Pakistan/AKU-21/2020 | 1 | 17187 | Syn | Orf1ab | helicase | p.5641L | c.16923ctA>ctG |
| hCov-19/Pakistan/AKU-37/2020 | 1 | 17815 | Nsyn | Orf1ab | helicase | p.5851G>R | c.17551Gga>Aga |
| hCov-19/Pakistan/AKU-57/2020 | 1 | 18318 | Syn | Orf1ab | 3'-to-5' exonuclease | p.6018G | c.18054ggG>ggT |
| hCov-19/Pakistan/AKU-50/2020 | 1 | 18462 | Syn | Orf1ab | 3'-to-5' exonuclease | p.6066P | c.18198ccG>ccT |
| hCoV19/Pakistan/JRCGRKHI17/2020 | 1 | 18508 | Nsyn | Orf1ab | 3'-to-5' exonuclease | p.6082L>F | c.18244Ctt>Ttt |
| PAK/NIH-45579/2020, hCoV19/Pakistan/NIH68/2020 | 2 | 18603 | Syn | Orf1ab | 3'-to-5' exonuclease | p.6113H | c.18339caT>caC |
| PAK/NIH-45579/2020 | 1 | 18788 | Nsyn | Orf1ab | 3'-to-5' exonuclease | p.6175T>I | c.18524aCa>aTa |
| hCov-19/Pakistan/AKU-2/2020, hCov-19/Pakistan/AKU-3/2020, hCov-19/Pakistan/AKU-16/2020, hCov-19/Pakistan/AKU-24/2020, hCov-19/Pakistan/AKU-25/2020, hCov-19/Pakistan/AKU-26/2020, hCov-19/Pakistan/AKU-27/2020, hCov-19/Pakistan/AKU-33/2020, hCov-19/Pakistan/AKU-37/2020, hCov-19/Pakistan/AKU-39/2020, hCov-19/Pakistan/AKU-46/2020, hCov-19/Pakistan/AKU-47/2020, hCov-19/Pakistan/AKU-54/2020, hCov-19/Pakistan/AKU-56/2020, hCov-19/Pakistan/AKU-66/2020, hCoV19/Pakistan/JRCGRKHI02/2020, hCoV19/Pakistan/JRCGRKHI03/2020, hCoV19/Pakistan/JRCGRKHI05/2020, hCoV19/Pakistan/JRCGRKHI06/2020, hCoV19/Pakistan/JRCGRKHI07/2020, hCoV19/Pakistan/JRCGRKHI11/2020, hCoV19/Pakistan/JRCGRKHI12/2020, hCoV19/Pakistan/JRCGRKHI13/2020, hCoV19/Pakistan/JRCGRKHI15/2020, hCoV19/Pakistan/JRCGRKHI16/2020, hCoV19/Pakistan/JRCGRKHI17/2020, hCoV19/Pakistan/JRCGRKHI06/2020, hCoV19/Pakistan/JRCGRKHI28/2020, hCoV19/Pakistan/JRCGRKHI29/2020, hCoV19/Pakistan/JRCGRKHI30/2020, hCoV19/Pakistan/JRCGRKHI33/2020, hCoV19/Pakistan/JRCGRKHI37/2020, hCoV19/Pakistan/JRCGRKHI40/2020, hCoV19/Pakistan/JRCGRKHI41/2020, hCoV19/Pakistan/JRCGRKHI42/2020, hCoV19/Pakistan/JRCGRKHI45/2020, hCoV19/Pakistan/JRCGRKHI47/2020, hCoV19/Pakistan/JRCGRKHI48/2020, hCoV19/Pakistan/NIBD01KHI/2020, hCoV19/Pakistan/NIBD02KHI/2020, hCoV19/Pakistan/NIH60/2020, hCoV19/Pakistan/NIH64/2020, hCoV-19/Pakistan/NIH-66/2020, hCoV-19/Pakistan/NIH-70/2020, hCoV-19/Pakistan/NIH-65/2020, hCoV-19/Pakistan/JRCGR-KHI35/2020 | 46 | 18877 | Syn | Orf1ab | 3'-to-5' exonuclease | p.6205L | c.18613Cta>Tta |
| hCoV19/Pakistan/NIBD01KHI/2020, hCoV19/Pakistan/NIBD02KHI/2020 | 2 | 19299 | Nsyn | Orf1ab | 3'-to-5' exonuclease | p.6345Y>* | c.19035taT>taG |
| hCoV19/Pakistan/JRCGRKHI03/2020 | 1 | 19401 | Syn | Orf1ab | 3'-to-5' exonuclease | p.6379S | c.19137tcT>tcC |
| hCov-19/Pakistan/AKU-67/2020 | 1 | 19572 | Syn | Orf1ab | 3'-to-5' exonuclease | p.6436Y | c.19308taC>taT |
| hCov-19/Pakistan/AKU-39/2020 | 1 | 19662 | Syn | Orf1ab | endoRNAse | p.6466H | c.19398caC>caT |
| hCov-19/Pakistan/AKU-56/2020 | 1 | 19677 | Nsyn | Orf1ab | endoRNAse | p.6471Q>H | c.19413caG>caT |
| hCov-19/Pakistan/AKU-57/2020 | 1 | 19891 | Nsyn | Orf1ab | endoRNAse | p.6543D>Y | c.19627Gat>Tat |
| hCoV-19/Pakistan/UN-UVAS-SIALKOT | 1 | 20031 | Syn | Orf1ab | endoRNAse | p.6589A | c.19767gcC>gcT |
| hCoV19/Pakistan/JRCGRKHI37/202 hCoV19/Pakistan/JRCGRKHI47/2020 0, hCoV-19/Pakistan/JRCGR-KHI35/2020 | 3 | 20062 | Nsyn | Orf1ab | endoRNAse | p.6600V>F | c.19798Gtt>Ttt |
| hCoV19/Pakistan/JRCGRKHI17/2020 | 1 | 20970 | Syn | Orf1ab | endoRNAse | p.6902V | c.20706gtC>gtG |
| hCov-19/Pakistan/AKU-37/2020, hCov-19/Pakistan/AKU-39/2020 | 2 | 20178 | Syn | Orf1ab | endoRNAse | p.6638V | c.19914gtC>gtT |
| hCov-19/Pakistan/AKU-54/2020, hCoV19/Pakistan/JRCGRKHI41/2020 | 2 | 21306 | Syn | Orf1ab | 2'-O-ribose methyltransferase | p.7014R | c.21042cgC>cgT |
| hCoV19/Pakistan/JRCGRKHI04/2020 | 1 | 21438 | Nsyn | Orf1ab | 2'-O-ribose methyltransferase | p.7058M>I | c.21174atG>atT |
| hCov-19/Pakistan/AKU-56/2020 | 1 | 21575 | Nsyn | S | S | p.5L>F | c.13Ctt>Ttt |
| hCoV-19/Pakistan/UN-UVAS-LAHORE-IV | 1 | 21784 | Nsyn | S | S | p.74N>K | c.222aaT>aaA |
| hCoV19/Pakistan/JRCGRKHI42/2020 | 1 | 21802 | Syn | S | S | p.80D | c.240gaT>gaC |
| hCov-19/Pakistan/AKU-66/2020 | 1 | 21786 | Nsyn | S | S | p.75G>V | c.224gGt>gTt |
| hCov-19/Pakistan/AKU-64/2020 | 1 | 21871 | Syn | S | S | p.103G | c.309ggC>ggT |
| hCov-19/Pakistan/AKU-25/2020 | 1 | 21974 | Nsyn | S | S | p.138D>Y | c.412Gat>Tat |
| hCoV19/Pakistan/NIH60/2020 | 1 | 22227 | Nsyn | S | S | p.222A>V | c.665gCt>gTt |
| hCov-19/Pakistan/AKU-24/2020, hCov-19/Pakistan/AKU-46/2020, hCov-19/Pakistan/AKU-47/2020, hCoV19/Pakistan/JRCGRKHI02/2020, hCoV19/Pakistan/JRCGRKHI17/2020, hCoV19/Pakistan/JRCGRKHI28/2020, hCoV19/Pakistan/JRCGRKHI29/2020, hCoV19/Pakistan/JRCGRKHI37/2020, hCoV19/Pakistan/JRCGRKHI47/2020, hCoV19/Pakistan/NIBD01KHI/2020 hCoV19/Pakistan/NIBD02KHI/2020, hCoV19/Pakistan/NIH64/2020, hCoV-19/Pakistan/JRCGR-KHI35/2020 | 13 | 22444 | Syn | S | S | p.294D | c.882gaC>gaT |
| hCov-19/Pakistan/AKU-10/2020, hCov-19/Pakistan/AKU-11/2020, hCoV19/Pakistan/JRCGRKHI04/2020 | 3 | 22468 | Syn | S | S | p.302T | c.906acG>acT |
| hCov-19/Pakistan/AKU-21/2020, hCov-19/Pakistan/AKU-67/2020, PAK/NIH-45579/2020, PAK/NIH-44090/2020, PAK/NIH-45143/2020, hCoV-19/Pakistan/KPK-KUST-SJTU/2020, hCoV19/Pakistan/JRCGRKHI22/2020, hCoV19/Pakistan/JRCGRKHI32/2020, hCoV19/Pakistan/NIH68/2020, hCoV-19/Pakistan/NIH-76/2020, hCoV19/Pakistan/NIH78/2020, hCoV19/Pakistan/NIH95/2020 | 12 | 22477 | Syn | S | S | p.305S | c.915tcC>tcT |
| hCoV-19/Pakistan/NIH-70/2020, hCoV-19/Pakistan/NIH-65/2020 | 2 | 23086 | Syn | S | S | p.508Y | C>T |
| hCov-19/Pakistan/AKU-4/2020 | 1 | 23129 | Nsyn | S | S | p.523T>A | c.1567Act>Gct |
| hCov-19/Pakistan/AKU-2/2020, hCov-19/Pakistan/AKU-3/2020, hCov-19/Pakistan/AKU-5/2020, hCov-19/Pakistan/AKU-16/2020, hCov-19/Pakistan/AKU-21/2020, hCov-19/Pakistan/AKU-24/2020, hCov-19/Pakistan/AKU-25/2020, hCov-19/Pakistan/AKU-26/2020, hCov-19/Pakistan/AKU-27/2020, hCov-19/Pakistan/AKU-33/2020, hCov-19/Pakistan/AKU-37/2020, hCov-19/Pakistan/AKU-39/2020, hCov-19/Pakistan/AKU-46/2020, hCov-19/Pakistan/AKU-47/2020, hCov-19/Pakistan/AKU-54/2020, hCov-19/Pakistan/AKU-56/2020, hCov-19/Pakistan/AKU-57/2020, hCov-19/Pakistan/AKU-64/2020, hCov-19/Pakistan/AKU-66/2020, hCov-19/Pakistan/AKU-67/2020, hCov-19/Pakistan/AKU-70/2020, PAK/NIH-45579/2020, PAK/NIH-44090/2020, PAK/NIH-45143/2020, hCoV-19/Pakistan/UN-UVAS-SIALKOT, hCoV-19/Pakistan/UN-UVAS-LAHORE-IV, hCoV-19/Pakistan/UN-UVAS-Lahore-III/2020, hCoV-19/Pakistan/UN-UVAS-Lahore-II/2020, hCoV-19/Pakistan/UN-UVAS-Lahore-IV/2020, hCoV-19/Pakistan/KPK-KUST-SJTU/2020, hCoV19/Pakistan/JRCGRKHI02/2020 , hCoV19/Pakistan/JRCGRKHI03/2020, hCoV19/Pakistan/JRCGRKHI05/2020, hCoV19/Pakistan/JRCGRKHI06/2020, hCoV19/Pakistan/JRCGRKHI07/2020, hCoV19/Pakistan/JRCGRKHI11/2020, hCoV19/Pakistan/JRCGRKHI12/2020, hCoV19/Pakistan/JRCGRKHI13/2020, hCoV19/Pakistan/JRCGRKHI15/2020, hCoV19/Pakistan/JRCGRKHI16/2020, hCoV19/Pakistan/JRCGRKHI17/2020, hCoV19/Pakistan/JRCGRKHI22/2020, hCoV19/Pakistan/JRCGRKHI06/2020, hCoV19/Pakistan/JRCGRKHI28/2020, hCoV19/Pakistan/JRCGRKHI29/2020, hCoV19/Pakistan/JRCGRKHI30/2020, hCoV19/Pakistan/JRCGRKHI32/2020, hCoV19/Pakistan/JRCGRKHI33/2020, hCoV19/Pakistan/JRCGRKHI37/2020, hCoV19/Pakistan/JRCGRKHI40/2020, hCoV19/Pakistan/JRCGRKHI41/2020, hCoV19/Pakistan/JRCGRKHI42/2020, hCoV19/Pakistan/JRCGRKHI45/2020, hCoV19/Pakistan/JRCGRKHI47/2020, hCoV19/Pakistan/JRCGRKHI48/2020, hCoV19/Pakistan/NIBD01KHI/2020, hCoV19/Pakistan/NIBD02KHI/2020, hCoV19/Pakistan/NIH60/2020, hCoV19/Pakistan/NIH64/2020, hCoV19/Pakistan/NIH68/2020, hCoV-19/Pakistan/NIH-76/2020, hCoV19/Pakistan/NIH78/2020, hCoV19/Pakistan/NIH95/2020, hCoV-19/Pakistan/NIH-66/2020, hCoV-19/Pakistan/NIH-70/2020, hCoV-19/Pakistan/NIH-62/2020, hCoV-19/Pakistan/NIH-65/2020, hCoV-19/Pakistan/JRCGR-KHI35/2020, hCoV-19/Pakistan/NIH-79/2020 | 69 | 23403 | Nsyn | S | S | p.614D>G | c.1841gAt>gGt |
| hCoV19/Pakistan/JRCGRKHI02/2020, hCoV19/Pakistan/NIH64/2020 | 2 | 23551 | Nsyn | S | S | p.663D | c.1989gaC>gaT |
| hCoV19/Pakistan/JRCGRKHI47/2020, hCoV-19/Pakistan/JRCGR-KHI35/2020 | 2 | 23593 | Nsyn | S | S | p.677Q>H | c.2031caG>caT |
| hCoV-19/Pakistan/UN-UVAS-LAHORE-IV, hCoV-19/Pakistan/UN-UVAS-Lahore-III/2020, hCoV-19/Pakistan/UN-UVAS-Lahore-II/2020, hCoV-19/Pakistan/UN-UVAS-Lahore-IV/2020, hCoV-19/Pakistan/NIH-62/2020, hCoV-19/Pakistan/NIH-79/2020 | 9 | 23731 | Syn | S | S | p.723T | c.2169acC>acT |
| hCov-19/Pakistan/AKU-23/2020, hCov-19/Pakistan/AKU-57/2020, hCoV-19/Pakistan/KP-RMI-01/2020 | 3 | 23929 | Syn | S | S | p.789Y | c.2367taC>taT |
| hCov-19/Pakistan/AKU-26/2020 | 1 | 24003 | Nsyn | S | S | p.814K>R | c.2441aAg>aGg |
| PAK/NIH-HAS001/2020, PAK/NIH-44905/2020 | 2 | 24051 | Nsyn | S | S | p.830D>A | c.2489gAt>gCt |
| hCov-19/Pakistan/AKU-25/2020, hCov-19/Pakistan/AKU-26/2020, hCov-19/Pakistan/AKU-33/2020, hCov-19/Pakistan/AKU-54/2020, hCov-19/Pakistan/AKU-56/2020, hCov-19/Pakistan/AKU-66/2020, hCoV19/Pakistan/JRCGRKHI06/2020, hCoV19/Pakistan/JRCGRKHI07/2020, hCoV19/Pakistan/JRCGRKHI11/2020, hCoV19/Pakistan/JRCGRKHI12/2020, hCoV19/Pakistan/JRCGRKHI15/2020, hCoV19/Pakistan/JRCGRKHI16/2020, hCoV19/Pakistan/JRCGRKHI06/2020, hCoV19/Pakistan/JRCGRKHI33/2020, hCoV19/Pakistan/JRCGRKHI40/2020, hCoV19/Pakistan/JRCGRKHI41/2020, hCoV19/Pakistan/JRCGRKHI42/2020, hCoV19/Pakistan/JRCGRKHI45/2020, hCoV19/Pakistan/JRCGRKHI48/2020, hCoV19/Pakistan/NIH60/2020 | 20 | 24202 | Syn | S | S | p.880G | c.2640ggT>ggC |
| hCoV19/Pakistan/JRCGRKHI04/2020, hCoV19/Pakistan/JRCGRKHI30/2020 | 2 | 24223 | Syn | S | S | p.887T | c.2661acC>acT |
| hCoV-19/Pakistan/UN-UVAS-SIALKOT | 1 | 24370 | Syn | S | S | p.936D | c.2808gaC>gaT |
| hCoV19/Pakistan/NIH60/2020 | 1 | 24355 | Syn | S | S | p.931I | c.2793atT>atA |
| hCoV19/Pakistan/JRCGRKHI03/2020, hCoV19/Pakistan/JRCGRKHI13/2020 | 2 | 24616 | Syn | S | S | p.1018I | c.3054atC>atT |
| hCov-19/Pakistan/AKU-66/2020 | 1 | 24757 | Syn | S | S | p.1065V | c.3195gtG>gtT |
| hCoV19/Pakistan/JRCGRKHI17/2020, hCoV19/Pakistan/JRCGRKHI28/2020, hCoV19/Pakistan/JRCGRKHI29/2020 | 3 | 25006 | Syn | S | S | p.1148F | c.3444ttC>ttT |
| hCoV19/Pakistan/JRCGRKHI40/2020 | 1 | 25020 | Nsyn | S | S | p.1153D>G | c.3458gAt>gGt |
| hCoV19/Pakistan/JRCGRKHI15/2020, hCoV19/Pakistan/JRCGRKHI45/2020 | 2 | 25046 | Nsyn | S | S | p.1162P>S | c.3484Cca>Tca |
| hCov-19/Pakistan/AKU-24/2020 | 1 | 25311 | Nsyn | S | S | p.1250C>F | c.3749tGt>tTt |
| hCoV19/Pakistan/JRCGRKHI32/2020, hCoV19/Pakistan/JRCGRKHI48/2020 | 2 | 25352 | Nsyn | S | S | p.1264V>L | c.3790Gtg>Ttg |
| hCoV19/Pakistan/JRCGRKHI37/2020 | 1 | 25393 | Nsyn | Orf3a | Orf3a | p.677Q>H | c.2031caG>caT |
| hCov-19/Pakistan/AKU-27/2020 | 1 | 25419 | Syn | Orf3a | Orf3a | p.9T | c.27acA>acG |
| hCoV19/Pakistan/NIH60/2020 | 1 | 25435 | Syn | Orf3a | Orf3a | p.15L | c.43Ttg>Ctg |
| hCoV19/Pakistan/JRCGRKHI42/2020 | 1 | 25455 | Nsyn | Orf3a | Orf3a | p.21K>N | c.63aaG>aaT |
| hCov-19/Pakistan/AKU-21/2020, hCov-19/Pakistan/AKU-2/2020, hCov-19/Pakistan/AKU-3/2020, hCov-19/Pakistan/AKU-5/2020, hCov-19/Pakistan/AKU-16/2020, hCov-19/Pakistan/AKU-24/2020, hCov-19/Pakistan/AKU-25/2020, hCov-19/Pakistan/AKU-26/2020, hCov-19/Pakistan/AKU-27/2020, hCov-19/Pakistan/AKU-33/2020, hCov-19/Pakistan/AKU-37/2020, hCov-19/Pakistan/AKU-39/2020, hCov-19/Pakistan/AKU-46/2020, hCov-19/Pakistan/AKU-47/2020, hCov-19/Pakistan/AKU-54/2020, hCov-19/Pakistan/AKU-56/2020, hCov-19/Pakistan/AKU-66/2020, hCov-19/Pakistan/AKU-67/2020, hCov-19/Pakistan/AKU-70/2020, hCov-19/Pakistan/AKU-51/2020, hCov-19/Pakistan/AKU-63/2020, PAK/NIH-45579/2020, PAK/NIH-44090/2020, PAK/NIH-45143/2020, hCoV-19/Pakistan/KPK-KUST-SJTU/2020, hCoV19/Pakistan/JRCGRKHI02/2020, hCoV19/Pakistan/JRCGRKHI03/2020, hCoV19/Pakistan/JRCGRKHI05/2020, hCoV19/Pakistan/JRCGRKHI06/2020, hCoV19/Pakistan/JRCGRKHI07/2020, hCoV19/Pakistan/JRCGRKHI11/2020, hCoV19/Pakistan/JRCGRKHI12/2020, hCoV19/Pakistan/JRCGRKHI13/2020, hCoV19/Pakistan/JRCGRKHI15/2020, hCoV19/Pakistan/JRCGRKHI16/2020, hCoV19/Pakistan/JRCGRKHI17/2020, hCoV19/Pakistan/JRCGRKHI18/2020, hCoV19/Pakistan/JRCGRKHI22/2020, hCoV19/Pakistan/JRCGRKHI06/2020, hCoV19/Pakistan/JRCGRKHI28/2020, hCoV19/Pakistan/JRCGRKHI29/2020, hCoV19/Pakistan/JRCGRKHI30/2020, hCoV19/Pakistan/JRCGRKHI32/2020, hCoV19/Pakistan/JRCGRKHI33/2020, hCoV19/Pakistan/JRCGRKHI37/2020, hCoV19/Pakistan/JRCGRKHI40/2020, hCoV19/Pakistan/JRCGRKHI41/2020, hCoV19/Pakistan/JRCGRKHI42/2020, hCoV19/Pakistan/JRCGRKHI45/2020, hCoV19/Pakistan/JRCGRKHI47/2020, hCoV19/Pakistan/JRCGRKHI48/2020, hCoV19/Pakistan/NIBD01KHI/2020, hCoV19/Pakistan/NIBD02KHI/2020, hCoV19/Pakistan/NIH60/2020, hCoV19/Pakistan/NIH64/2020, hCoV19/Pakistan/NIH68/2020, hCoV-19/Pakistan/NIH-76/2020, hCoV19/Pakistan/NIH78/2020, hCoV19/Pakistan/NIH95/2020, hCoV-19/Pakistan/NIH-66/2020, hCoV-19/Pakistan/NIH-70/2020, hCoV-19/Pakistan/NIH-65/2020, hCoV-19/Pakistan/JRCGR-KHI35/2020 | 63 | 25563 | Nsyn | Orf3a | Orf3a | p.57Q>H | c.171caG>caT |
| hCov-19/Pakistan/AKU-33/2020 | 1 | 25572 | Syn | Orf3a | Orf3a | p.60S | c.180tcC>tcT |
| hCov-19/Pakistan/AKU-39/2020 | 1 | 25685 | Nsyn | Orf3a | Orf3a | p.98A>V | c.293gCt>gTt |
| PAK/NIH-45143/2020 | 1 | 25706 | Nsyn | Orf3a | Orf3a | p.105F>S | c.314tTt>tCt |
| hCov-19/Pakistan/AKU-37/2020, hCov-19/Pakistan/AKU-39/2020 | 2 | 25855 | Nsyn | Orf3a | Orf3a | p.155D>Y | c.463Gac>Tac |
| hCoV-19/Pakistan/KHI1/2020 | 1 | 26022 | Syn | Orf3a | Orf3a | p.210D | c.630gaC>gaT |
| hCov-19/Pakistan/AKU-54/2020, hCoV19/Pakistan/JRCGRKHI41/2020 | 2 | 26060 | Nsyn | Orf3a | Orf3a | p.223T>I | c.668aCt>aTt |
| hCov-19/Pakistan/AKU-58/2020, hCov-19/Pakistan/AKU-59/2020 | 2 | 26263 | Nsyn | E | E | p.7E>K | c.19Gaa>Aaa |
| PAK/NIH-HAS001/2020, PAK/NIH-44905/2020 | 2 | 26313 | Syn | E | E | p.23F | c.69ttC>ttT |
| hCoV19/Pakistan/JRCGRKHI07/2020, hCoV19/Pakistan/JRCGRKHI15/2020, hCoV19/Pakistan/JRCGRKHI30/2020, hCoV19/Pakistan/JRCGRKHI42/2020, hCoV19/Pakistan/JRCGRKHI45/2020 | 5 | 26534 | Syn | M | M | p.4S | c.12tcC>tcT |
| hCoV-19/Pakistan/NIH-70/2020, hCoV-19/Pakistan/NIH-65/2020 | 2 | 26677 | Nsyn | M | M | p.52I>S | T>G |
| hCoV19/Pakistan/JRCGRKHI42/2020 | 1 | 26690 | Syn | M | M | p.56L | c.168ctG>ctT |
| hCov-19/Pakistan/AKU-2/2020, hCov-19/Pakistan/AKU-3/2020, hCov-19/Pakistan/AKU-16/2020, hCov-19/Pakistan/AKU-24/2020, hCov-19/Pakistan/AKU-25/2020, hCov-19/Pakistan/AKU-26/2020, hCov-19/Pakistan/AKU-27/2020, hCov-19/Pakistan/AKU-33/2020, hCov-19/Pakistan/AKU-37/2020, hCov-19/Pakistan/AKU-39/2020, hCov-19/Pakistan/AKU-46/2020, hCov-19/Pakistan/AKU-47/2020, hCov-19/Pakistan/AKU-54/2020, hCov-19/Pakistan/AKU-56/2020, hCov-19/Pakistan/AKU-66/2020, hCov-19/Pakistan/AKU-52/2020, hCoV19/Pakistan/JRCGRKHI02/2020, hCoV19/Pakistan/JRCGRKHI03/2020, hCoV19/Pakistan/JRCGRKHI05/2020, hCoV19/Pakistan/JRCGRKHI07/2020, hCoV19/Pakistan/JRCGRKHI11/2020, hCoV19/Pakistan/JRCGRKHI12/2020, hCoV19/Pakistan/JRCGRKHI13/2020, hCoV19/Pakistan/JRCGRKHI15/2020, hCoV19/Pakistan/JRCGRKHI16/2020, hCoV19/Pakistan/JRCGRKHI17/2020, hCoV19/Pakistan/JRCGRKHI18/2020, hCoV19/Pakistan/JRCGRKHI28/2020, hCoV19/Pakistan/JRCGRKHI29/2020, hCoV19/Pakistan/JRCGRKHI30/2020, hCoV19/Pakistan/JRCGRKHI33/2020, hCoV19/Pakistan/JRCGRKHI37/2020, hCoV19/Pakistan/JRCGRKHI40/2020, hCoV19/Pakistan/JRCGRKHI41/2020, hCoV19/Pakistan/JRCGRKHI42/2020, hCoV19/Pakistan/JRCGRKHI45/2020, hCoV19/Pakistan/JRCGRKHI47/2020, hCoV19/Pakistan/JRCGRKHI48/2020, hCoV19/Pakistan/NIBD01KHI/2020, hCoV19/Pakistan/NIBD02KHI/2020, hCoV19/Pakistan/NIH60/2020, hCoV19/Pakistan/NIH64/2020, hCoV-19/Pakistan/NIH-66/2020, hCoV-19/Pakistan/NIH-70/2020, hCoV-19/Pakistan/NIH-65/2020, hCoV-19/Pakistan/JRCGR-KHI35/2020 | 46 | 26735 | Syn | M | M | p.71Y | c.213taC>taT |
| hCoV19/Pakistan/JRCGRKHI37/2020, hCoV19/Pakistan/JRCGRKHI47/2020, hCoV-19/Pakistan/JRCGR-KHI35/2020 | 3 | 26895 | Nsyn | M | M | p.125H>Y | c.373Cat>Tat |
| hCoV19/Pakistan/JRCGRKHI04/2020 | 1 | 26912 | Syn | M | M | p.130T | c.390acC>acA |
| hCov-19/Pakistan/AKU-25/2020 | 1 | 26947 | Nsyn | M | M | p.142A>V | c.425gCt>gTt |
| hCov-19/Pakistan/AKU-46/2020 | 1 | 27068 | Syn | M | M | p.182G | c.546ggA>ggC |
| hCoV19/Pakistan/JRCGRKHI32/2020 | 1 | 27046 | Nsyn | M | M | p.175T>M | c.524aCg>aTg |
| hCoV19/Pakistan/NIH60/2020 | 1 | 27059 | Syn | M | M | p.179Y | c.537taC>taT |
| hCov-19/Pakistan/AKU-25/2020 | 1 | 27204 | Nsyn | Orf6 | Orf6 | p.1M>I | c.3atG>atT |
| hCoV19/Pakistan/JRCGRKHI02/2020, hCoV19/Pakistan/NIH64/2020 | 2 | 27297 | Syn | Orf6 | Orf6 | p.32I | c.96atC>atT |
| hCoV19/Pakistan/JRCGRKHI15/2020, hCoV19/Pakistan/JRCGRKHI45/2020 | 2 | 27331 | Nsyn | Orf6 | Orf6 | p.44L>V | c.130Cta>Gta |
| hCoV19/Pakistan/JRCGRKHI17/2020 | 1 | 27384 | Syn | Orf6 | Orf6 | p.61D | c.183gaT>gaC |
| hCov-19/Pakistan/AKU-56/2020 | 1 | 27442 | Nsyn | Orf7 | Orf7 | p.17L>F | c.49Ctt>Ttt |
| hCov-19/Pakistan/AKU-52/2020 | 1 | 27549 | Syn | Orf7 | Orf7 | p.52N | c.156aaC>aaT |
| hCov-19/Pakistan/AKU-52/2020 | 1 | 27621 | Nsyn | Orf7 | Orf7 | p.76Q>H | c.228caG>caT |
|  |  |  |  |  |  |  |  |
|  |  |  |  |  |  |  |  |
|  |  |  |  |  |  |  |  |
| hCov-19/Pakistan/AKU-47/2020 | 1 | 27995 | Syn | Orf8 | Orf8 | p.34D | c.102gaT>gaC |
| PAK/NIH-44090/2020, hCoV19/Pakistan/JRCGRKHI41/2020 | 2 | 28027 | Syn | Orf8 | Orf8 | p.45W>L | c.134tGg>tTg |
| hCov-19/Pakistan/AKU-10/2020, hCov-19/Pakistan/AKU-11/2020, PAK/NIH-HAS001/2020, PAK/NIH-44905/2020, hCoV19/Pakistan/JRCGRKHI04/2020 | 5 | 28144 | Nsyn | Orf8 | Orf8 | p.84L>S | c.251tTa>tCa |
| PAK/NIH-HAS001/2020, PAK/NIH-44905/2020 | 2 | 28167 | Nsyn | Orf8 | Orf8 | p.92E>K | c.274Gaa>Aaa |
| hCov-19/Pakistan/AKU-24/2020 | 1 | 28182 | Nsyn | Orf8 | Orf8 | p.97S>G | c.289Agt>Ggt |
| hCov-19/Pakistan/AKU-65/2020 | 1 | 28240 | Nsyn | Orf8 | Orf8 | p.116V>A | c.347gTt>gCt |
| hCoV-19/Pakistan/NIH-62/2020, hCoV-19/Pakistan/NIH-65/2020, hCoV-19/Pakistan/NIH-79/2020 | 3 | 28250 | Nsyn | Orf8 | Orf8 | p.119L | insCTG |
| hCoV-19/Pakistan/KP-RMI-01/2020 | 1 | 28253 | Syn | Orf8 | Orf8 | p.120F | c.360ttC>ttT |
| hCov-19/Pakistan/AKU-21/2020 | 1 | 28310 | Nsyn | N | N | p.13P>T | c.37Ccc>Acc |
| hCov-19/Pakistan/AKU-23/2020, hCoV-19/Pakistan/KP-RMI-01/2020 | 2 | 28311 | Nsyn | N | N | p.13P>L | c.38cCc>cTc |
| hCov-19/Pakistan/AKU-47/2020 | 1 | 28354 | Nsyn | N | N | p.27N | c.81aaC>aaT |
| PAK/NIH-45579/2020, hCoV19/Pakistan/NIH68/2020 | 1 | 28378 | Syn | N | N | p.35A | c.105gcG>gcC |
| hCoV19/Pakistan/JRCGRKHI32/2020 | 1 | 28392 | Nsyn | N | N | p.40R>L | c.119cGt>cTt |
| hCov-19/Pakistan/AKU-65/2020 | 1 | 28407 | Nsyn | N | N | p.45L>S | c.134tTa>tCa |
| hCoV19/Pakistan/JRCGRKHI33/2020 | 1 | 28606 | Syn | N | N | p.111Y | c.333taC>taT |
| hCov-19/Pakistan/AKU-58/2020, hCov-19/Pakistan/AKU-59/2020 | 2 | 28688 | Syn | N | N | p.139L | c.415Ttg>Ctg |
| hCov-19/Pakistan/AKU-58/2020, hCov-19/Pakistan/AKU-59/2020 | 2 | 28709 | Nsyn | N | N | p.139L | c.415Ttg>Ctg |
| hCov-19/Pakistan/AKU-65/2020 | 1 | 28733 | Nsyn | N | N | p.154N>D | c.460Aat>Gat |
| hCoV-19/Pakistan/UN-UVAS-SIALKOT | 1 | 28810 | Syn | N | N | p.179G | c.537ggC>ggT |
| hCov-19/Pakistan/AKU-23/2020 | 1 | 28812 | Nsyn | N | N | p.180S>I | c.539aGt>aTt |
| hCov-19/Pakistan/AKU-37/2020, hCov-19/Pakistan/AKU-39/2020 | 2 | 28824 | Nsyn | N | N | p.184S>Y | c.551tCt>tAt |
| hCov-19/Pakistan/AKU-24/2020, hCov-19/Pakistan/AKU-46/2020, hCov-19/Pakistan/AKU-47/2020, hCov-19/Pakistan/AKU-52/2020, hCoV19/Pakistan/JRCGRKHI02/2020, hCoV19/Pakistan/JRCGRKHI17/2020, hCoV19/Pakistan/JRCGRKHI28/2020, hCoV19/Pakistan/JRCGRKHI29/2020, hCoV19/Pakistan/JRCGRKHI37/2020, hCoV19/Pakistan/JRCGRKHI47/2020, hCoV19/Pakistan/NIBD01KHI/2020, hCoV19/Pakistan/NIBD02KHI/2020, hCoV19/Pakistan/NIH64/2020, hCoV-19/Pakistan/JRCGR-KHI35/2020 | 14 | 28854 | Nsyn | N | N | p.194S>L | c.581tCa>tTa |
| hCoV-19/Pakistan/NIH-70/2020, hCoV-19/Pakistan/NIH-65/2020 | 2 | 28865 | Nsyn | N | N | p.198T>A | A>G |
| hCov-19/Pakistan/AKU-10/2020, hCov-19/Pakistan/AKU-11/2020, PAK/NIH-HAS001/2020, PAK/NIH-45579/2020, PAK/NIH-44905/2020, hCoV19/Pakistan/JRCGRKHI04/2020, hCoV19/Pakistan/NIH68/2020 | 7 | 28878 | Nsyn | N | N | p.202S>N | c.605aGt>aAt |
| hCov-19/Pakistan/AKU-64/2020, hCoV-19/Pakistan/UN-UVAS-SIALKOT, hCoV-19/Pakistan/UN-UVAS-LAHORE-IV, hCoV-19/Pakistan/UN-UVAS-Lahore-III/2020, hCoV-19/Pakistan/UN-UVAS-Lahore-II/2020, hCoV-19/Pakistan/UN-UVAS-Lahore-IV/2020, hCoV-19/Pakistan/NIH-62/2020, hCoV-19/Pakistan/NIH-79/2020 | 8 | 28881 | Nsyn | N | N | p.203R>K | c.608aGg>aAg |
| hCov-19/Pakistan/AKU-64/2020, hCoV-19/Pakistan/UN-UVAS-SIALKOT, hCoV-19/Pakistan/UN-UVAS-LAHORE-IV, hCoV-19/Pakistan/UN-UVAS-Lahore-III/2020, hCoV-19/Pakistan/UN-UVAS-Lahore-II/2020, hCoV-19/Pakistan/UN-UVAS-Lahore-IV/2020, hCoV-19/Pakistan/NIH-62/2020, hCoV-19/Pakistan/NIH-79/2020 | 8 | 28882 | Syn | N | N | p.203R | c.609agG>agA |
| hCov-19/Pakistan/AKU-64/2020, hCoV-19/Pakistan/UN-UVAS-SIALKOT, hCoV-19/Pakistan/UN-UVAS-LAHORE-IV, hCoV-19/Pakistan/UN-UVAS-Lahore-III/2020, hCoV-19/Pakistan/UN-UVAS-Lahore-II/2020, hCoV-19/Pakistan/UN-UVAS-Lahore-IV/2020, hCoV-19/Pakistan/NIH-62/2020, hCoV-19/Pakistan/NIH-79/2020 | 9 | 28883 | Nsyn | N | N | p.204G>R | c.610Gga>Cga |
| hCoV-19/Pakistan/KP-RMI-01/2020 | 1 | 28887 | Nsyn | N | N | p.205T>I | c.614aCt>aTt |
| hCov-19/Pakistan/AKU-25/2020, hCov-19/Pakistan/AKU-26/2020, hCov-19/Pakistan/AKU-33/2020, hCov-19/Pakistan/AKU-54/2020, hCov-19/Pakistan/AKU-56/2020, hCov-19/Pakistan/AKU-66/2020, hCoV19/Pakistan/JRCGRKHI07/2020, hCoV19/Pakistan/JRCGRKHI12/2020, hCoV19/Pakistan/JRCGRKHI15/2020, hCoV19/Pakistan/JRCGRKHI16/2020, hCoV19/Pakistan/JRCGRKHI30/2020, hCoV19/Pakistan/JRCGRKHI33/2020, hCoV19/Pakistan/JRCGRKHI40/2020, hCoV19/Pakistan/JRCGRKHI41/2020, hCoV19/Pakistan/JRCGRKHI42/2020, hCoV19/Pakistan/JRCGRKHI45/2020, hCoV19/Pakistan/JRCGRKHI48/2020, hCoV19/Pakistan/NIH60/2020 | 18 | 28899 | Nsyn | N | N | p.209R>I | c.626aGa>aTa |
| hCov-19/Pakistan/AKU-46/2020, hCoV-19/Pakistan/NIH-76/2020, hCoV19/Pakistan/NIH78/2020, hCoV19/Pakistan/NIH95/2020 | 4 | 28975 | Nsyn | N | N | p.234M>I | c.702atG>atT |
| hCov-19/Pakistan/AKU-26/2020, hCov-19/Pakistan/AKU-33/2020, hCov-19/Pakistan/AKU-54/2020, hCov-19/Pakistan/AKU-66/2020, hCov-19/Pakistan/AKU-50/2020, hCoV19/Pakistan/JRCGRKHI40/2020, hCoV19/Pakistan/JRCGRKHI41/2020, hCoV19/Pakistan/JRCGRKHI48/2020 | 8 | 29215 | Syn | N | N | p.314F | c.942ttC>ttT |
| hCoV-19/Pakistan/KPK-KUST-SJTU/2020, hCoV19/Pakistan/JRCGRKHI22/2020 | 2 | 29253 | Nsyn | N | N | p.327S>L | c.980tCg>tTg |
| hCov-19/Pakistan/AKU-21/2020 | 1 | 29383 | Nsyn | N | N | p.370K>N | c.1110aaG>aaT |
| hCoV19/Pakistan/JRCGRKHI17/2020 | 1 | 29409 | Nsyn | N | N | p.379T>I | c.1136aCt>aTt |
| hCov-19/Pakistan/AKU-56/2020 | 1 | 29468 | Nsyn | N | N | p.399D>Y | c.1195Gat>Tat |
| hCoV19/Pakistan/JRCGRKHI17/2020 | 1 | 29598 | Nysn | Orf10 | Orf10 | p.14Y>C | c.41tAt>tGt |
| hCoV-19/Pakistan/KP-RMI-01/2020, hCoV-19/Pakistan/KPK-KUST-SJTU/2020 | 2 | 29645 | Nsyn | Orf10 | Orf10 | p.30V>L | c.88Gta>Tta |
| PAK/NIH-44090/2020 | 1 | 29696 | - | 3'UTR | 3'UTR | - | C > T |
| hCov-19/Pakistan/AKU-47/2020 | 1 | 29708 | - | 3'UTR | 3'UTR | - | T > C |
| hCov-19/Pakistan/AKU-46/2020 | 1 | 29729 | - | 3'UTR | 3'UTR | - | T > C |
| hCoV-19/Pakistan/NIH-62/2020 | 1 | 29730 | - | 3'UTR | 3'UTR |  | C>T |
| hCov-19/Pakistan/AKU-27/2020 | 1 | 29738 | - | 3'UTR | 3'UTR | - | T > C |
| hCov-19/Pakistan/AKU-10/2020, hCov-19/Pakistan/AKU-11/2020, AKU-Cov_58, AKU-Cov_59, PAK/NIH-HAS001/2020, PAK/NIH-44905/2020, hCoV19/Pakistan/JRCGRKHI04/2020 | 7 | 29742 | - | 3'UTR | 3'UTR | - | G > A |
| hCoV-19/Pakistan/NIH-70/2020 | 1 | 29772 | - | 3'UTR | 3'UTR | - | T>A |
| hCoV-19/Pakistan/NIH-65/2020 | 1 | 29779 | - | 3'UTR | 3'UTR | - | G>T |
| hCov-19/Pakistan/AKU-27/2020 | 1 | 29747 | - | 3'UTR | 3'UTR | - | T > G |
| hCov-19/Pakistan/AKU-46/2020, hCoV19/Pakistan/JRCGRKHI02/2020 | 2 | 29755 | - | 3'UTR | 3'UTR | - | G > T |
| hCoV-19/Pakistan/JRCGR-KHI35/2020 | 1 | 29802 | - | 3'UTR | 3'UTR | - | C>A |
| hCoV-19/Pakistan/NIH-79/2020 | 1 | 29812 | - | 3'UTR | 3'UTR | - | A>T |
| hCoV19/Pakistan/JRCGRKHI12/2020 | 1 | 29836 | - | 3'UTR | 3'UTR | - | CCCATGTGATTTTAATAGCTTCTTAGGAGAATGACAAAAAAAAAAAAAAAAAAAAAAAAAAAAAAAA > C |
| hCoV19/Pakistan/JRCGRKHI30/2020 | 1 | 29838 | - | 3'UTR | 3'UTR | - | C > T |
| hCoV19/Pakistan/JRCGRKHI13/2020 | 1 | 29870 | - | 3'UTR | 3'UTR | - | C > A |
| hCoV-19/Pakistan/UN-UVAS-SIALKOT | 1 | 29865 | - | 3'UTR | 3'UTR | - | G > A |

UTR: untranslated region; 3CLPro: 3C like protease; E: envelope protein; 3’-5’ exonuclease; M: membrane glycoprotein; N: nucleocaspid phosphoprotein; NA: not applicable; NSP: non-structural protein; OMT: O-methyltransferase; ORF: open reading frame; RdRp: RNA-dependent RNA polymerase; SARS-CoV-2: severe acute respiratory syndrome coronavirus 2; S: spike glycoprotein; UTR: untranslated region. Syn: synonymous, Nsyn: non =-synonymous.
