# Supplementary material for: Higher entropy observed in SARS-CoV-2 genomes from the first COVID-19 wave in Pakistan
Source: PLoS One. 2021 Aug 31;16(8):e0256451. doi: 10.1371/journal.pone.0256451 (PMC8407562; doi:10.1371/journal.pone.0256451)
Supplement: Attachment — Submitted filename: Response_to reviewers_30_7_21.docx [file pone.0256451.s009.docx]

**View Letter**

Dear Dr. Ata,

Editor PLOS ONE,

Thank you for the review of our submitted manuscript:

PONE-D-21-09819
Increased diversity with absence of site-specific selection bias in SAR-CoV-2 genomes from the early pandemic period in Pakistan.

We provide responses to the questions raised by the reviewers as below:

Journal Requirements:

When submitting your revision, we need you to address these additional requirements.

*1. Please ensure that your manuscript meets PLOS ONE's style requirements, including those for file naming. The PLOS ONE style templates can be found at*

*https://journals.plos.org/plosone/s/file?id=wjVg/PLOSOne_formatting_sample_main_body.pdf and*

[*https://journals.plos.org/plosone/s/file?id=ba62/PLOSOne_formatting_sample_title_authors_affiliations.pdf*](about:blank)

-	thank you for sharing these links. We have revised the manuscript in accordance with the required style templates

*2.Thank you for including your ethics statement:  "This work received approval by the Ethical Review Committee, Aga Khan University.".*

*Please provide additional details regarding participant consent. In the ethics statement in the Methods and online submission information, please ensure that you have specified (1) whether consent was informed and (2) what type you obtained (for instance, written or verbal, and if verbal, how it was documented and witnessed). If your study included minors, state whether you obtained consent from parents or guardians. If the need for consent was waived by the ethics committee, please include this information.*

*If you are reporting a retrospective study of medical records or archived samples, please ensure that you have discussed whether all data were fully anonymized before you accessed them and/or whether the IRB or ethics committee waived the requirement for informed consent. If patients provided informed written consent to have data from their medical records used in research, please include this information*

*Once you have amended this/these statement(s) in the Methods section of the manuscript, please add the same text to the “Ethics Statement” field of the submission form (via “Edit Submission”).*

*For additional information about PLOS ONE ethical requirements for human subjects research, please refer to http://journals.plos.org/plosone/s/submission-guidelines#loc-human-subjects-research.*

 -	thank you for this comment. We have described the process of testing from anonymized archived samples and Ethical Review Committee approval was received as a wavier of informed consent for the project. This has been added in the Methods section.

*3.In your Data Availability statement, you have not specified where the minimal data set underlying the results described in your manuscript can be found. PLOS defines a study's minimal data set as the underlying data used to reach the conclusions drawn in the manuscript and any additional data required to replicate the reported study findings in their entirety. All PLOS journals require that the minimal data set be made fully available. For more information about our data policy, please see http://journals.plos.org/plosone/s/data-availability.*

*Upon re-submitting your revised manuscript, please upload your study’s minimal underlying data set as either Supporting Information files or to a stable, public repository and include the relevant URLs, DOIs, or accession numbers within your revised cover letter. For a list of acceptable repositories, please see http://journals.plos.org/plosone/s/data-availability#loc-recommended-repositories. Any potentially identifying patient information must be fully anonymized.*

- All data is available publically. Accession numbers of the SARS-CoV-2 deposited and available are present in Methods and also in Supplementary Table 1.

Important: If there are ethical or legal restrictions to sharing your data publicly, please explain these restrictions in detail. Please see our guidelines for more information on what we consider unacceptable restrictions to publicly sharing data: http://journals.plos.org/plosone/s/data-availability#loc-unacceptable-data-access-restrictions. Note that it is not acceptable for the authors to be the sole named individuals responsible for ensuring data access.

- Not applicable – data is provided

We will update your Data Availability statement to reflect the information you provide in your cover letter.

*4. Please amend your list of authors on the manuscript to ensure that each author is linked to an affiliation. Authors’ affiliations should reflect the institution where the work was done (if authors moved subsequently, you can also list the new affiliation stating “current affiliation:….” as necessary).*

- This correction has been made.

Reviewers' comments:

Reviewers Comments to the Author

Reviewer #1: In this manuscript, 32 SARS-CoV-2 strains were isolated, of which 21 were complete genome and 11 partial. Complete genome sequences have been submitted to Genbank. Phylogenic, SNP and selection analyses have been performed for these genomes.

*Comments:
(1) This manuscript was not well-organized and the English writings need to be improved, such as typos and misuse of punctuation.*

- We have re-organised the paper and made necessary English language corrections.

*(2) There should be more details about SARS-CoV-2 cases, epidemiology and evolution in Pakistan in t-he background part.*

-	The background has been revised to include more details about COVID-19 in Pakistan and to also include what is know about the genomic epidemiology of SARS-CoV-2 strains from the country.

*(3) There are 57 complete and high coverage genome sequences available from Pakistan at Sep. 31, 2000 in the GISAID EpiCoV database. These genomes need to be added for phylogenetic analyses. Moreover, other genome sequences used in analyses need description. E.g., the choose of the 449 full-length sequences, and the 6523 global complete sequences. By the way, the "supplemtary file 1" is not available.*

-	We thank the reviewer for pointing this out. We have now included all additional samples available from Pakistan on GISAID. Therefore, we have conducted analysis of 90 genomes in total.

Supplementary file 1 has been added – we apologise that it was missed last time. Corrections have been made to correctly list the number of strains which were analysed.

*(4) HyPHY SLAC is not adequate for selection pressure analysis. More methods need to be applied. And the gene/cds used for the analysis need to be indicated.
 -*	Thank you for the advice. Additional analysis has been conducted and included in the study. These are described in methods and the results. Further, statistical analysis has been performed.

*(5) High-resolution figures for Fig. 1 and 2 need to be provided. Phylogenetic tree should be displayed in rectangular, rather than circular.
-*	Fig 1 phylogenetic tree has been displayed as a rectangular figure. High-resolution figures have now been included.

*(6) A clear conclusion need be provided. The difference and relationship of first and second wave should be discussed.*
-	Conclusions have been revised and the relationships between the first and second wave of COVID-19 in Pakistan have been discussed.

*(7) Some references were not cited properly.*

-	References have been checked and cited as necessary.

*Reviewer #2: This manuscript presents a meaningful and useful result, and futher increases our understanding of SAR-CoV-2 in Pakistan.
However, the genomic analysis of this manuscript is not deep enough.*

*For example, in Fig1 and Fig2, the clades and isolate date are needed to shown in figures and analysis, the additional analysis may make the conclusion of this manuscript more credible.*

*Page16: Figure 3.*

- The comments of Reviewer 2 are not completely clear. We understand that the reviewer required a deeper genomic analysis. This has now been provided through the additional analysis that has been conducted.
- Further we have included additional figures to describe the clades as per the date that they were identified (S Fig1 and S Fig 2).
- Further mutational analysis has been performed regarding the entropy across the genome and also site-specific entropy. The conclusions have been justified accordingly and we hope that this will not be acceptable
